# miRNome profile in blood samples upstream and downstream of the coronary lesion and arterial aortic root before and after angioplasty in subjects with chronic and acute coronary syndrome: A pilot observational study protocol (Plaque study)

**DOI:** 10.1371/journal.pone.0324467

**Published:** 2025-06-13

**Authors:** Olga Protic, Anna Rita Bonfigli, Fabiola Olivieri, Adrianapia Maria Lamedica, Gabriele Gabrielli, Roberto Antonicelli

**Affiliations:** 1 Scientific Direction, IRCCS INRCA, Ancona, Italy; 2 Advanced Technology Center for Aging Research, IRCCS INRCA, Ancona, Italy; 3 Department of Clinical and Molecular Sciences, Università Politecnica delle Marche, Ancona, Italy; 4 Interventional Cardiology Unit, IRCCS INRCA, Ancona, Italy; 5 Cardiology Unit, IRCCS INRCA, Ancona, Italy; UN Mehta Institute of Cardiology and Research Center, INDIA

## Abstract

Currently the mechanisms that lead an atherosclerotic plaque to become unstable and those that trigger the coagulative/thrombotic processes leading to acute coronary syndrome have yet to be clarified. It has been suggested a possible role of microRNAs in the physiopathology of the atherosclerotic process related to acute and chronic ischemic cardiomyopathy. However, no data exists on the correlation between microRNAs expression in coronary (upstream and downstream of the coronary lesion) and arterial (at the aortic root level) blood from patients with acute coronary syndromes (ACS) and chronic coronary syndromes (CCS) before and after angioplasty. The study’s primary objective is to assess miRnome analysis in coronary and arterial blood sampling in ACS and CCS patients before and after angioplasty. The secondary objective is to analyze interleukin-6 and soluble ST2 levels in the peripheral plasma samples before and after angioplasty. Ten patients with ACS and ten patients with CCS will be enrolled. Coronary stenosis treated with angioplasty will be located in the proximal segments of the three main vessels: the anterior interventricular artery, the circumflex artery, and the right coronary artery. The angioplasty procedure will be performed according to standard clinical practice. Before and after angioplasty, blood samples upstream and downstream of the coronary lesion will be taken, arterial sampling at the aortic root level will be performed, and peripheral venous blood will be collected. The expression of serum microRNAs will be analyzed by Next-generation sequencing. Quantitative analysis of pro- and anti-inflammatory molecules such as interleukin-6 and the soluble form of the soluble ST2 will be performed on various blood samples. This study is registered in ClinicalTrials.Gov on October 30, 2023 (ID NCT06103357).

## Introduction

Acute Myocardial Infarction (AMI) is the principal clinical presentation of coronary artery disease (CAD) [1]. Despite decades of studies of AMI pathophysiology, many aspects remain to be clarified. In particular, mechanisms that lead an atherosclerotic plaque to become unstable and those that trigger coagulative/thrombotic processes leading to acute coronary syndrome (ACS). ACS is a broad term for conditions such as Non-ST-Elevation Myocardial Infarction (NSTEMI), ST-elevation myocardial infarction (STEMI), and Unstable Angina [2]. The most accepted pathophysiological hypotheses maintain that AMI is related to plaque rupture or erosion of vulnerable plaque [3], and inflammation was hypothesized to play a role in this context [4,5]. Atherosclerotic plaque formation is an inflammatory process in the endothelial vessel associated with low-density lipoprotein retention (LDL) [6]. Some potential biomarkers from atherosclerotic plaques have been described [7].

miRNAs are small regulatory RNAs involved in different biological processes and pathologies and could be efficient biomarkers for diseases. The role of miRNAs in regulating different biological pathways involved in the development and progression of atherosclerosis has been found using preclinical models [8]. In humans, altered expression of several circulating miRNAs has been identified in the presence of stable atherosclerotic plaques [9]. Our group previously identified the circulating miRNAs involved in modulating the inflammatory state, the inflamma-miRs, and miRNAs associated with cardiomyocyte necrosis (miR-499) [10,11]. However, no published data exists on the correlation between microRNA expression in coronary, arterial, and peripheral venous blood from patients with acute (ACS) or chronic (CCS) coronary syndromes.

During ACS, the plaque becomes unstable, triggering biological processes that lead to partial or complete vessel thrombosis. Many of these mechanisms remain unclear and it has been shown that miRNAs are involved [12–15]. In this study we aim to investigate whether there are differences in the expression of miRNAs before and after the activation of an unstable plaque. Specifically, we aim to compare these findings with the miRNA profile in a stable plaque associated with CCS. In this way, this study will investigate if there is a different expression of miRNAs downstream of the unstable plaque, differing from the levels present before the plaque activation, which leads to the acute event.

It is known that some miRNAs are involved in cellular regeneration [16], thrombotic processes [17], and activating inflammatory pathways [11]. miRNome analysis from different blood samples of ACS and CCS patients could reveal the mechanisms involved in the pathogenesis of AMI and bring new input to clinical and therapeutic perspectives.

This study will enroll patients affected by ACS and CCS with clinical indications for coronary angiography, demonstrating the presence of significant coronary artery disease. Three types of samples will be collected, before and after angioplasty. From peripheral venous blood, samples upstream and downstream of the coronary lesion, and samples at the aortic root level.

Blood flow before the coronary lesion could reflect more systemic conditions, such as inflammatory status and endothelial dysfunction. Downstream regions of the coronary lesions are characterized by lower oxygenation and nutrient flow due to the restricted blood flow, which could alter the miRNAs profile. Changes in miRNA levels downstream of a coronary lesion may be due to unregulated secretion from injured/stressed cells.

This study aims to test the miRNome picture in the blood of subjects undergoing an angioplasty procedure according to standard clinical practice. The expression of circulating miRNAs will be analyzed by Next-generation sequencing (NGS). In addition to blood sampling, where possible, plaque fragments will be taken to highlight differences in the biomarkers examined, particularly the immune/inflammatory profile. In addition to miRNome analysis, this protocol will include the quantitative analysis of pro and anti-inflammatory molecules such as interleukin-6 (IL-6) [18,19] and the soluble form of the interleukin-33 receptor (sST2) [20–22]. IL-33/sST2 axis is involved in the modulation of the inflammatory response since sST2 exerts proinflammatory effects when secreted into the circulation. Previous studies demonstrated that elevation of serum sST2 levels was associated with poor prognosis not only in patients with heart failure (HF) but also with myocardial infarction (MI), hypothesizing that elevated serum sST2 level might be closely related to vulnerable plaque features [23]. Recently it was hypothesized that elevated serum sST2 levels might be closely related to vulnerable plaque features, serving as a simple biomarker for coronary plaque vulnerability [24].

## Methods

### Study design

**Fig 1:**
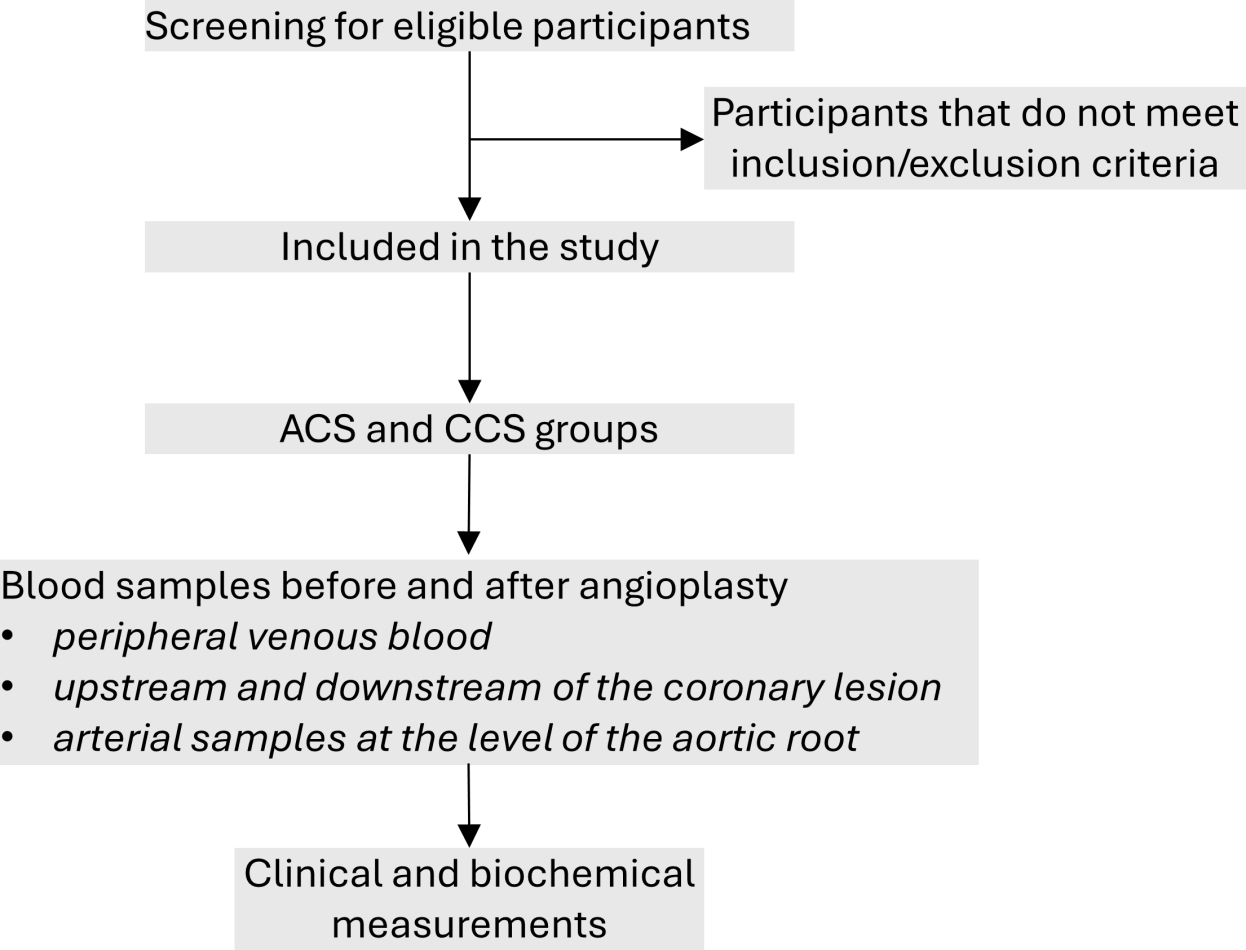
Schematic representation of the study design.

This protocol (Version 1.0_15 December 2022) has been approved by the Ethics Committee of the IRCCS INRCA (reference ID: CE-INRCA-22032). This work describes a pilot observational study protocol. Ten patients with NSTEMI ACS and ten patients with SCC with clinical indication and favorable anatomy for coronary angioplasty at the Interventional Cardiology Unit, IRCCS INRCA, Ancona, Italy, will be enrolled in this study. Inclusion/exclusion criteria will be applied. Patients who meet the inclusion criteria and wish to participate in the trial must sign a written informed consent presented by a physician. A schematic representation of the study design is shown in (Fig 1).

### Objectives

#### Primary objective.

To compare miRNome profile in coronary and arterial blood samples between ACS and CCS patients before and after angioplasty.

#### Secondary objective.

To compare levels of biomarkers of inflammation such as IL-6 and sST2 in plasma samples between ACS and CCS subjects before and after angioplasty.

### Inclusion/exclusion criteria

#### Inclusion criteria.

- Age > 18 years;
- Signed written informed consent;
- Clinical indication for the percutaneous coronary intervention (PCI) procedure according to the European Society of Cardiology (ESC) Guidelines [25];
- Coronary stenosis treated with angioplasty must be located in the proximal segments of the three main vessels: the anterior interventricular artery, the circumflex artery, and the right coronary artery;
- The vessels must have a diameter of their mid-distal section ≥ 3 mm;
- For ACS group: patients with NSTEMI ACS and clinical indication with favorable anatomy for PCI;
- For CCS group: patients with clinically incipient CCS with stable angina (or significant anginal equivalents) and clinical indication with favorable anatomy for PCI.

#### Exclusion criteria.

- Contraindications to anticoagulant/antiplatelet therapy;
- Extensive calcifications and/or tortuosity of the major epicardial segments;
- Evidence of thrombotic occupation;
- Patients with hemodynamic instability;
- Patients with EF (ejection fraction) < 35%;
- Patients with severe chronic renal failure (e-GFR < 30 mL/min).

### Patient screening and recruitment

During the screening phase, information on the of the subject’s health status (diagnosis, medical history, and pharmacological therapies), socio-demographic data will be taken, and anthropometric parameters will be measured. In addition, participants will undergo blood tests according to the profile described in Table 1. The patient’s clinical data will be collected during the care pathway through an electronic medical record. The trial is opened for patient enrollment and recruitment on 06 March 2023.

**Table 1:** Study schedule.

	Screening	ACS group		CCS group	
		Before angioplasty	After angioplasty	Before angioplasty	After angioplasty
Informed consent	X				
Recording demographic data	X				
Medical history	X				
Pharmacological therapy	X				
Anthropometric measurements	X				
Fool blood exam-FBE^1^	X				
Leukocytes formula^2^	X				
Renal function^3^	X				
Liver function^4^	X				
Lipid profile^5^	X				
Electrolytes^6^	X				
Fasting glucose	X				
C-reactive protein	X				
Erythrocyte sedimentation rate	X				
High-sensitivity cardiac troponin	X				
Uric acid	X				
Prothrombin time	X				
Interleukin-6	X	X	X	X	X
sST2^7^	X	X	X	X	X
miRNome		Blood samples upstream and downstream of the coronary lesion	Blood samples upstream and downstream of the coronary lesion	Arterial sampling at the aortic root level	Arterial sampling at the aortic root level

ACS = acute coronary syndrome; CCS = chronic coronary syndrome;

^1^White blood cells (WBCs), red blood cells (RBCs), hemoglobin, hematocrit, mean cell volume (MCV), mean corpuscular hemoglobin (MCH), mean corpuscular hemoglobin concentration (MCHC), red cell distribution width (RDW), platelets;

^2^Neutrophils, eosinophils, basophils, lymphocytes, monocytes;

^3^Creatinine; estimated glomerular filtration rate by CKD-EPI equation;

^4^Aspartate aminotransferase glutamic oxaloacetic transaminase (AST/GOT), alanine aminotransferase/ glutamic pyruvic transaminase (ALT/GPT);

^5^Total cholesterols, triglyceride, low-density cholesterol (LDL), and high-density cholesterol (HDL);

^6^Sodium, potassium, magnesium;

^7^Soluble form of the IL-33 receptor.

### Coronary angiography/angioplasty procedure and conservation of plaque samples

Coronary stenosis treated with angioplasty needs to be located in the proximal segments of the three main vessels: the anterior interventricular artery, the circumflex artery, and the right coronary artery.

The coronary angiography will be performed with radial or femoral arterial access, using introducers and 6 or 7 F catheters, and with a Siemens Artis angiography system (Siemens Healthineers, Forchheim, Germany). Intracoronary ultrasonography (IVUS), which is routinely performed in hospital INRCA, Ancona, Italy, in case of complex angioplasties, will also be carried out using the Philips Core Mobile system, which will allow the evaluation of the plaque both with the grayscale and with the virtual histology. The angioplasty procedure will be performed according to standard clinical practice. Blood samples and, when possible, fragments of plaque/thrombus in proximity (< 10 mm, upstream and downstream) of the coronary lesion will be taken before and after angioplasty using the Pronto LP 5 F microcatheter (Teleflex Inc. USA). The distal protection system used to collect plaque fragments during the interventional procedure will be the Spider FX Embolic Protection System (Medtronic Europe). The system consists of a microcatheter with a guide wire at the end at which point a nitinol basket of variable size is fixed. At the beginning of the interventional procedure, it will be positioned distal to the lesion to be examined. The filter’s patency, will allow the collection of plaque fragments that may dislodge during the procedure without interfering with the blood supply to the vessel’s periphery. At the end of the procedure, the filter will be removed and the plaque fragments will be taken. The material collected in the basket of the Spider Fx device will be carefully removed from the filter, collected in Falcon-type test tubes, and frozen at -80° C for future analysis at the Hospital INRCA laboratory, Ancona, Italy. Samples at the aortic root level will be drawn using a JR4 Super Torque Plus 5 F diagnostic catheter (Cordis Inc. USA) and venous samples from peripheral blood.

### Blood samples

Before and after angioplasty, the following blood samples will be taken:

- Peripheral venous blood
- Upstream and downstream of the coronary lesion
- Arterial samples at the level of the aortic root

Blood samples will be centrifuged, plasma and serum samples will be aliquoted and frozen at -80°C until further analysis.

### Biomarkers analyses

To identify the miRNome picture, the Next Generation Sequencing (NGS) techniques will be used.

The quantitative analyses of IL-6 and sST2 will be performed by standard procedures.

### Outcomes

1. Difference in miRNome profile in blood samples upstream and downstream of the coronary lesion and arterial samples at the level of the aortic root between ACS and CCS subjects;
2. Difference in pro- and anti-inflammatory molecules such as IL-6 and sST2 levels in peripheral bloods samples before and after angioplasty.

### Statistical analysis

The expression values of circulating miRNAs and pro- and anti-inflammatory molecules in arterial and venous blood samples will be compared by paired measures tests before and after the interventional procedure. Furthermore, the levels of biomarkers between the two groups of patients (ACS and CCS) for the various types of sampling will be compared by tests for the comparison between independent samples. A value of p < 0.05 will be considered statistically significant.

### Ethics and dissemination

The study will follow Good Clinical Practice (GCP), the ethical principles derived from the Declaration of Helsinki, and current legislation on observational studies.

- All potentially eligible subjects will receive complete information on the study and provide written consent;
- Participants will be informed that they can leave the study anytime without specifying a reason;
- Participants must consent to the processing of personal data in anonymous and aggregate form according to EU Regulation 2016/679 (GDPR);
- The subject will be informed and asked to provide an ad hoc informed consent to participate in the study, which includes data retention for up to 15 years after the study’s conclusion;
- The data collected will be kept strictly confidential, and a database will be set up at the Cardiology Unit, Ancona, Italy. In compliance with the protection regulations, data entry and processing of data will be accessible only to the personnel of the Cardiology Unit involved in the study via password;
- Participants will be informed that their data may be reviewed by authorized personnel or members of the relevant ethics committee and officials of the relevant regulatory authorities;
- Study findings will be presented at scientific conferences and submitted for publication in peer-reviewed journals.

### Management and reporting of adverse events/adverse reactions

Due to the observational nature of this study adverse events/reactions except those that may occur during standard clinical practice procedures are not expected.

### Dissemination plan and results communication

The study’s supervisor will write a final report and make the results public at the end of the study. The data will be made public anonymously and presented as required in aggregated mode.

## Discussion

Cardiovascular diseases (CVDs) lead to high prevalence of mortality and are an important global health problem [26]. Myocardial infarction is the leading cause of death in developed countries [27]. On the other hand, CVDs have a heavy impact on economic burden [28]. It is worth investing time and resources in research that will bring new insights into the pathophysiology of myocardial infarction. MiRNAs could be novel potential prognostic biomarkers and contribute to a deeper understanding of the etiopathology of AMI.

This study protocol is designed to examine the levels of innovative biomarkers involved in the pathogenesis of AMI in subjects with ACS and CCS. The protocol does not include any particular change in clinical procedures compared to the standard clinical management of patients affected by ACS and SCC with clinical indications for coronary angiography. The study follows GCP and the performance of the routine coronagraphic examination. The sampling of the biological material involves a brief extension in the times of the standard procedure. However, before stepping into large sample-size studies, it is necessary to examine the feasibility of performing coronary blood sampling from ACS and SCC patients.

Our group strongly supports the transparency of publishing the data in CVD research regardless of the outcome or significance of findings [29,30]. A patient’s willingness to participate in this kind of study is relevant. It may give a new insight into understanding the physiopathology of AMI, which is still the most common cause of mortality worldwide.

MiRNAs play a crucial role in gene regulation and have been implicated in various diseases, including AMI. To our knowledge, no published data exists on the correlation between miRNAs expression in coronary, arterial, and peripheral venous blood from patients with ACS or CCS. miRNome analysis from different blood samples of ACS and CCS patients could contribute to clarify the molecular mechanisms involved in the pathogenesis of AMI and bring new input to clinical and therapeutic perspectives. A limitation of this pilot study is the limited sample size of 20 patients. Within this small sample size, variability might be an important issue. To overcome this limitation, further study designs should consider the following: maximize the timing between non-STEMI symptoms and PCI laboratory assessment, since limited inflammation might be observed after 48 hours [25]; infarct size should be limited as this introduces a large inflammatory response [31]; intravascular ultrasound IVUS characteristics of plaque vulnerability/rupture/erosion should be performed [32]. Further studies with a larger number of patients will be necessary to verify the expression levels of specific miRNA in coronary, arterial and peripheral venous blood before and after angioplasty.

## Supporting information

S1 File SPIRIT checklist. (DOCX)

S2 File Plaque project. (PDF)

S3 File Study protocol. (PDF)

## References

[1] Thygesen K, Alpert JS, White HD, Joint ESC/ACCF/AHA/WHF Task Force for the Redefinition of Myocardial Infarction. Universal definition of myocardial infarction. J Am Coll Cardiol. 2007;50(22):2173–95. doi: 10.1016/j.jacc.2007.09.011 18036459

[2] Anderson JL, Adams CD, Antman EM, Bridges CR, Califf RM, Casey DE Jr, et al. 2012 ACCF/AHA focused update incorporated into the ACCF/AHA 2007 guidelines for the management of patients with unstable angina/non-ST-elevation myocardial infarction: a report of the American College of Cardiology Foundation/American Heart Association Task Force on Practice Guidelines. Circulation. 2013;127(23):e663–828. doi: 10.1161/CIR.0b013e31828478ac 23630129

[3] Arbab-Zadeh A, Fuster V. From detecting the vulnerable plaque to managing the vulnerable patient: JACC state-of-the-art review. J Am Coll Cardiol. 2019;74(12):1582–93. doi: 10.1016/j.jacc.2019.07.062 31537269

[4] Maier W, Altwegg LA, Corti R, Gay S, Hersberger M, Maly FE, et al. Inflammatory markers at the site of ruptured plaque in acute myocardial infarction: locally increased interleukin-6 and serum amyloid A but decreased C-reactive protein. Circulation. 2005;111(11):1355–61. doi: 10.1161/01.CIR.0000158479.58589.0A 15753219

[5] Antonicelli R, Olivieri F, Cavallone L, Spazzafumo L, Bonafè M, Marchegiani F, et al. Tumor necrosis factor-alpha gene -308G>A polymorphism is associated with ST-elevation myocardial infarction and with high plasma levels of biochemical ischemia markers. Coron Artery Dis. 2005;16(8):489–93. doi: 10.1097/00019501-200512000-00006 16319659

[6] Kobiyama K, Ley K. Atherosclerosis. Circ Res. 2018;123(10):1118–20. doi: 10.1161/CIRCRESAHA.118.313816 30359201 PMC6298754

[7] Adam CA, Șalaru DL, Prisacariu C, Marcu DTM, Sascău RA, Stătescu C. Novel biomarkers of atherosclerotic vascular disease-latest insights in the research field. Int J Mol Sci. 2022;23(9):4998. doi: 10.3390/ijms23094998 35563387 PMC9103799

[8] Andreou I, Sun X, Stone PH, Edelman ER, Feinberg MW. miRNAs in atherosclerotic plaque initiation, progression, and rupture. Trends Mol Med. 2015;21(5):307–18. doi: 10.1016/j.molmed.2015.02.003 25771097 PMC4424146

[9] Pereira-da-Silva T, Napoleão P, Costa MC, Gabriel AF, Selas M, Silva F, et al. Circulating miRNAs are associated with the systemic extent of atherosclerosis: novel observations for miR-27b and miR-146. Diagnostics (Basel). 2021;11(2):318. doi: 10.3390/diagnostics11020318 33669374 PMC7920287

[10] Olivieri F, Antonicelli R, Spazzafumo L, Santini G, Rippo MR, Galeazzi R, et al. Admission levels of circulating miR-499-5p and risk of death in elderly patients after acute non-ST elevation myocardial infarction. Int J Cardiol. 2014;172(2):e276-8. doi: 10.1016/j.ijcard.2013.12.203 24461971

[11] Olivieri F, Prattichizzo F, Giuliani A, Matacchione G, Rippo MR, Sabbatinelli J, et al. miR-21 and miR-146a: the microRNAs of inflammaging and age-related diseases. Ageing Res Rev. 2021;70:101374. doi: 10.1016/j.arr.2021.101374 34082077

[12] Rozhkov AN, Shchekochikhin DY, Ashikhmin YI, Mitina YO, Evgrafova VV, Zhelankin AV, et al. The profile of circulating blood microRNAs in outpatients with vulnerable and stable atherosclerotic plaques: associations with cardiovascular risks. Noncoding RNA. 2022;8(4):47. doi: 10.3390/ncrna8040047 35893230 PMC9326687

[13] Hajibabaie F, Kouhpayeh S, Mirian M, Rahimmanesh I, Boshtam M, Sadeghian L, et al. MicroRNAs as the actors in the atherosclerosis scenario. J Physiol Biochem. 2020;76(1):1–12. doi: 10.1007/s13105-019-00710-7 31808077

[14] Huang P. Potential new therapeutic targets: association of microRNA with atherosclerotic plaque stability. Int J Immunopathol Pharmacol. 2023;37:3946320231185657. doi: 10.1177/03946320231185657 37403558 PMC10331098

[15] Koroleva IA, Nazarenko MS, Kucher AN. Role of microRNA in development of instability of atherosclerotic plaques. Biochemistry (Mosc). 2017;82(11):1380–90. doi: 10.1134/S0006297917110165 29223165

[16] Pezhouman A, Nguyen NB, Kay M, Kanjilal B, Noshadi I, Ardehali R. Cardiac regeneration - Past advancements, current challenges, and future directions. J Mol Cell Cardiol. 2023;182:75–85. doi: 10.1016/j.yjmcc.2023.07.009 37482238

[17] Thibord F, Munsch G, Perret C, Suchon P, Roux M, Ibrahim-Kosta M, et al. Bayesian network analysis of plasma microRNA sequencing data in patients with venous thrombosis. Eur Heart J Suppl. 2020;22(Suppl C):C34–45. doi: 10.1093/eurheartj/suaa008 32368197 PMC7189740

[18] Sano M. Complexity of inflammation in the trajectory of vascular disease: interleukin 6 and beyond. Ann Vasc Dis. 2023;16(1):8–16. doi: 10.3400/avd.ra.23-00003 37006867 PMC10064308

[19] Antonicelli R, Olivieri F, Bonafè M, Cavallone L, Spazzafumo L, Marchegiani F, et al. The interleukin-6 -174 G>C promoter polymorphism is associated with a higher risk of death after an acute coronary syndrome in male elderly patients. Int J Cardiol. 2005;103(3):266–71. doi: 10.1016/j.ijcard.2004.08.064 16098388

[20] Thanikachalam PV, Ramamurthy S, Mallapu P, Varma SR, Narayanan J, Abourehab MA, et al. Modulation of IL-33/ST2 signaling as a potential new therapeutic target for cardiovascular diseases. Cytokine Growth Factor Rev. 2023;71–72:94–104. doi: 10.1016/j.cytogfr.2023.06.003 37422366

[21] Sciatti E, Merlo A, Scangiuzzi C, Limonta R, Gori M, D’Elia E, et al. Prognostic value of sST2 in heart failure. J Clin Med. 2023;12(12):3970. doi: 10.3390/jcm12123970 37373664 PMC10299183

[22] Sabbatinelli J, Giuliani A, Bonfigli AR, Ramini D, Matacchione G, Campolucci C, et al. Prognostic value of soluble ST2, high-sensitivity cardiac troponin, and NT-proBNP in type 2 diabetes: a 15-year retrospective study. Cardiovasc Diabetol. 2022;21(1):180. doi: 10.1186/s12933-022-01616-3 36088327 PMC9463761

[23] Jenkins WS, Roger VL, Jaffe AS, Weston SA, AbouEzzeddine OF, Jiang R, et al. Prognostic Value of soluble ST2 after myocardial infarction: a community perspective. Am J Med. 2017;130(9):1112.e9-1112.e15. doi: 10.1016/j.amjmed.2017.02.034 28344136 PMC5572469

[24] Luo G, Qian Y, Sheng X, Sun J, Wu Z, Liao F, et al. Elevated serum levels of soluble ST2 are associated with plaque vulnerability in patients with Non-ST-elevation acute coronary syndrome. Front Cardiovasc Med. 2021;8:688522. doi: 10.3389/fcvm.2021.688522 34368249 PMC8341076

[25] Neumann F-J, Sousa-Uva M, Ahlsson A, Alfonso F, Banning AP, Benedetto U, et al. 2018 ESC/EACTS guidelines on myocardial revascularization. Eur Heart J. 2019;40(2):87–165. doi: 10.1093/eurheartj/ehy394 30165437

[26] Amini M, Zayeri F, Salehi M. Trend analysis of cardiovascular disease mortality, incidence, and mortality-to-incidence ratio: results from global burden of disease study 2017. BMC Public Health. 2021;21(1):401. doi: 10.1186/s12889-021-10429-0 33632204 PMC7905904

[27] Fox KAA, Steg PG, Eagle KA, Goodman SG, Anderson FA Jr, Granger CB, et al. Decline in rates of death and heart failure in acute coronary syndromes, 1999-2006. JAMA. 2007;297(17):1892–900. doi: 10.1001/jama.297.17.1892 17473299

[28] Luengo-Fernandez R, Walli-Attaei M, Gray A, Torbica A, Maggioni AP, Huculeci R, et al. Economic burden of cardiovascular diseases in the European Union: a population-based cost study. Eur Heart J. 2023;44(45):4752–67. doi: 10.1093/eurheartj/ehad583 37632363 PMC10691195

[29] Bonfigli AR, Protic O, Olivieri F, Montesanto A, Malatesta G, Di Pillo R, et al. Effects of a novel nutraceutical combination (BruMeChol™) in subjects with mild hypercholesterolemia: study protocol of a randomized, double-blind, controlled trial. Trials. 2020;21(1):616. doi: 10.1186/s13063-020-04551-4 32631422 PMC7336431

[30] Protic O, Di Pillo R, Montesanto A, Galeazzi R, Matacchione G, Giuliani A, et al. Randomized, double-blind, placebo-controlled trial to test the effects of a nutraceutical combination monacolin K-free on the lipid and inflammatory profile of subjects with hypercholesterolemia. Nutrients. 2022;14(14):2812. doi: 10.3390/nu14142812 35889769 PMC9324786

[31] Frangogiannis NG. The inflammatory response in myocardial injury, repair, and remodelling. Nat Rev Cardiol. 2014;11(5):255–65. doi: 10.1038/nrcardio.2014.28 24663091 PMC4407144

[32] Mishra B, Pandit AK, Miyachi S, Ohshima T, Kawaguchi R, Vishnu VY, et al. Clinical utility of intravascular ultrasound (IVUS) in carotid artery interventions: a systematic review and meta-analysis. J Endovasc Ther. 2022;29(5):678–91. doi: 10.1177/15266028211064824 34955053

