# Supplementary material for: miRNome profile in blood samples upstream and downstream of the coronary lesion and arterial aortic root before and after angioplasty in subjects with chronic and acute coronary syndrome: A pilot observational study protocol (Plaque study)
Source: PLoS One. 2025 Jun 13;20(6):e0324467. doi: 10.1371/journal.pone.0324467 (PMC12165368; doi:10.1371/journal.pone.0324467)
Supplement: S2 File — (PDF) [file pone.0324467.s002.pdf]

PROPOSAL FORM

Instructions: Please use font type Times New Roman, font 12, interline spacing 1, text adjusted.

**Section A**	**GENERAL INFORMATION**
**NETWORK**<br>*[double-click 1 box, select 'checked']*	☐ Rete Alleanza Contro il Cancro<br>☐ Rete Neurologica<br>**X** Rete Cardiologica<br>☐ Rete IDEA<br>☐ Rete Aging<br>☐ Rete RAMS
Legal Representative Network	*Dr. Lorenzo Menicanti*
Project Leader: IRCCS	*Centro Cardiologico Monzino IRCCS*
Legal Representative IRCCS leader	*Ing. Mauro Melis*
Point of Contact (Name, e-mail, tel.)	*Dr.ssa Francesca Colazzo, francesca.colazzo@cardiologicomonzino.it; +39 0258002369*

RESEARCH PROGRAM DESCRIPTION

**Research Program Title**	***Atherosclerosis and Ischemic Heart Disease***
**Abstract**	Ischemic heart disease (IHD) represents the main cause of morbidity and mortality in industrialized countries. This pathology includes all conditions with an insufficient supply of blood and oxygen to the heart and is primarily determined by atherosclerosis, a degenerative multifactorial disease. The activities of the programme are mainly focused on the study of acute myocardial infarction and coronary heart disease and their clinical *sequelae*. In particular, studies are underway to assess predictors of left ventricular remodelling and elucidate the cellular and molecular components involved in the atherosclerotic process. Specifically, the VavirimS and CoPhyTeA studies aim to identify early predictors of left ventricular (LV) remodelling and reperfusion injury, respectively, which are associated with poor prognosis in patients with ST-segment elevation myocardial infarction (STEMI). Furthermore, the Plaque project "Integrated strategies for the study of cellular and genomic determinants of atheromatous plaque" seeks to identify neuro-humoral, immunological, cellular, and transcriptional markers involved in the pathogenesis of the atherosclerotic lesions. The results of these programs will provide the basis for the development of new therapeutic approaches. In particular, the results of the first two projects will contribute to better early risk stratification of patients with STEMI and a better understanding of the mechanisms underlying LV remodelling and reperfusion injury. Studies on atherosclerosis will address issues ranging from the pathophysiology of the disease to the clinical correlates of atherosclerotic plaques: the project aims to bridge the knowledge gap in coronary and carotid atherosclerotic disease between the molecular and cellular factors involved and clinical presentation and outcome, addressing several unmet needs. The overall results may help design personalized therapeutic strategies to prevent/treat the onset/development of IHD.
**Keywords** (max. 5)	1. acute myocardial infarction<br>2. coronary heart disease<br>3. left ventricle remodelling<br>4. atherosclerosis<br>5. carotid atherosclerotic disease
**Main, measurable research products and activities**	Within the "***Atherosclerosis and Ischemic Heart Disease Programme***", the following projects are activated -<br>1. The **VavirimS study**, coordinated by San Raffaele Hospital, evaluates the predictive value of myocardial viability measured by cardiac magnetic resonance

	imaging (MRI) in identifying adverse LV remodelling six months after a STEMI event in patients undergoing successful primary percutaneous coronary intervention (PPCI). The study also evaluates the diagnostic accuracy of cardiac MRI at one month in predicting LV remodelling at six months by estimating the technique's sensitivity, specificity, and negative predictive value. The VavirimS study is considered the first significant application area of the Cardiology Network node within the Health Big Data project. Testing tools for textual analysis of clinical reports related to the project is planned.<br>2. The **CoPhyTeA study** is an observational, prospective, multicenter study of patients with STEMI undergoing PPCI that aims to establish the predictive value of coronary physiology indices measured invasively by a special catheter immediately after PPCI on myocardial tissue characterization assessed by cardiac MRI within one week after the acute event. The results of these studies will provide important information for early risk stratification of patients with STEMI and may contribute to a better understanding of the mechanisms underlying LV remodelling and reperfusion injury with potential therapeutic impact.<br>3. The **Plaque Project** aims to identify determinants of atherosclerosis, integrating investigations in different research areas to address issues ranging from pathophysiology to the clinical correlates of atherosclerotic plaque. In particular, the project will focus on two topics, coronary atherosclerosis and carotid atherosclerosis, and related conditions.
**Scientific output**	Where applicable, data collection within the program is carried out through the data platform of the Cardiology Network (https://retecardiologica.cbim.it/redcap/index.php), a customized software solution based on the REDCap web application used by all IRCCS Associates, allowing the storage of large volumes of data. Implementation of the platform is ongoing to allow the storage and the consultation of DICOM (Digital Imaging and COmmunications in Medicine) images. To this end, the central repository of images will be managed by a software platform capable to be interfaced with and acquiring images in a vendor-independent and multi-site way.<br><br>Below are the publications obtained from the program:<br>*1.* Hall IF et al. rs41291957 controls miR-143 and miR-145 expression and impacts coronary artery disease risk. EMBO Mol Med. 2021;13(10):e14060. Epub 2021/09/23. doi: 10.15252/emmm.202114060. PMID: 34551209.<br>*2.* Carbone F et al. Serum levels of osteopontin predict major adverse cardiovascular events in patients with severe carotid artery stenosis. Int J Cardiol. 2018;255:195-9. Epub 2018/01/11. doi: 10.1016/j.ijcard.2018.01.008. PMID: 29317141.<br>*3.* Liberale L et al. Serum adiponectin levels predict acute coronary syndrome (ACS) in patients with severe carotid stenosis. Vascul Pharmacol. 2018;102:37-43. Epub 2018/01/07. doi: 10.1016/j.vph.2017.12.066. PMID: 29305337.<br>*4.* Liberale L et al. Resistin exerts a beneficial role in atherosclerotic plaque inflammation by inhibiting neutrophil migration. Int J Cardiol. 2018;272:13-9. Epub 2018/08/05. doi: 10.1016/j.ijcard.2018.07.112. PMID: 30075966.<br>*5.* Liberale L et al. Serum PCSK9 levels predict the occurrence of acute coronary syndromes in patients with severe carotid artery stenosis. Int J Cardiol. 2018;263:138-41. Epub 2018/05/15. doi: 10.1016/j.ijcard.2018.03.081. PMID: 29754909.<br>*6.* Rigamonti F et al. Serum lipoprotein (a) predicts acute coronary syndromes in patients with severe carotid stenosis. Eur J Clin Invest. 2018;48(3). Epub 2018/01/13. doi: 10.1111/eci.12888. PMID: 29327345.<br>*7.* da Silva RF et al. Anti-Apolipoprotein A-1 IgG Influences Neutrophil Extracellular Trap Content at Distinct Regions of Human Carotid Plaques. Int J Mol Sci. 2020;21(20). Epub 2020/10/23. doi: 10.3390/ijms21207721. PMID: 33086507.<br>*8.* Bonaventura A et al. Platelet-to-lymphocyte ratio at the time of carotid endarterectomy is associated with acute coronary syndrome occurrence. J Cardiovasc Med (Hagerstown).

2020;21(1):80-2. Epub 2019/10/01. doi: 10.2459/JCM.0000000000000869. PMID: 31567635.

9. Carbone F et al. Ficolin-2 serum levels predict the occurrence of acute coronary syndrome in patients with severe carotid artery stenosis. Pharmacol Res. 2021;166:105462. Epub 2021/01/30. doi: 10.1016/j.phrs.2021.105462. PMID: 33513354.
10. Chiesa M et al. Whole-blood transcriptome profile at hospital admission discriminates between patients with ST-segment elevation and non-ST-segment elevation acute myocardial infarction. Sci Rep 2020;10:8731. doi: 10.1038/s41598-020- 65527-7. PMID: 32457432.
11. Vavassori C et al. Circulating microRNAs as novel biomarkers in risk assessment and prognosis of coronary artery disease. Eur Cardiol Rev 2022, in press. doi: 10.15420/ecr.2021.47.
12. Arbustini E et al. On the Shades of Coronary Calcium and Plaque Instability. J Am Coll Cardiol. 2021;77(13):1612-5. Epub 2021/04/03. doi: 10.1016/j.jacc.2021.02.011. PMID: 33795034.
13. Prati F et al. Potential of an Approach Based on the Identification and Treatment of Vulnerable Coronary Plaques. JACC Cardiovasc Interv. 2021;14(4):468-73. Epub 2021/02/20. doi: 10.1016/j.jcin.2020.12.033. PMID: 33602444.
14. Chaudhry F et al. Molecular Imaging of Apoptosis in Atherosclerosis by Targeting Cell Membrane Phospholipid Asymmetry. J Am Coll Cardiol. 2020;76(16):1862- 74. Epub 2020/10/17. doi: 10.1016/j.jacc.2020.08.047. PMID: 33059832.

**IRCCS INVOLVED**	*Number 20*
1.	IRCCS Centro Cardiologico Monzino
2.	IRCCS Fondazione Policlinico San Matteo
3.	IRCCS Istituti Clinici Scientifici Maugeri S.p.A. Società Benefit
4.	IRCCS Istituto Clinico Humanitas
5.	IRCCS Istituto di Ricerche Farmacologiche Mario Negri
6.	IRCCS San Raffaele Roma
7.	ISMETT Istituto Mediterraneo per i Trapianti e Terapie ad Alta Specializzazione
8.	IRCCS NEUROMED
9.	IRCCS Fondazione Istituto Neurologico Nazionale C. Mondino
10.	IRCCS Ospedale Policlinico San Martino
11.	IRCCS Ospedale San Raffaele
12.	IRCCS MultiMedica
13.	Fondazione Policlinico Universitario Agostino Gemelli IRCCS
14.	IRCCS Istituto Auxologico Italiano
15.	IRCCS Policlinico San Donato
16.	IRCCS Fondazione Ca' Granda - Ospedale Maggiore Policlinico
17.	IRCCS SDN Synlab
18.	IRCCS Istituto Nazionale di Riposo e Cura per Anziani - INRCA
19.	Istituto Giannina Gaslini IRCCS
20.	Ospedale Pediatrico Bambino Gesù

**Start Date and duration**	**2018 - *2025***
**TOTAL AMOUNT OF FUNDS RECEIVED FROM THE MOH FOR THE PROGRAM**	**Following the found received from the MOH €**

**PROJECT**	**FUNDED PROJECT CODE**	**€**
VavirimS study	RRC-2018-2365810	**179.000,00**
CoPhyTeA study	RCR-2019-23669118_003	**442.000,00**
Plaque Project	RRC-2018-2365810<br>RCR-2019-23669118_001	**1.137.100,00**

Other Sources of Funds	*source of funding*	*(€*

**Scientific program coordinator**	Prof. Paolo Camici<br>March 5th 1949

AFFILIATION<br>INSTITUTION DEPARTMENT/UNIT	Ospedale San Raffaele IRCCS, Cardiovascular Research Centre
POSITION TITLE	Professor of Cardiology and Director of the Cardiovascular Research Centre
TELEPHONE AND E-MAIL	Tel/Fax: +39 0226436206/6218<br>Email: camici.paolo@hsr.it

PERSONAL STATEMENT & short presentation of the Scientific program coordinator

Professor Camici graduated in Medicine and Surgery at the University of Pisa and specialized in Cardiology and Nuclear Medicine. He moved to the UK in 1991 as a Professor of Cardiology at Imperial College School of Medicine. In 2010, he was appointed Professor of Cardiology at Vita-Salute University and San Raffaele Hospital, Milan, IT. He is the Director of the CardioCenter at San Raffaele Hospital. His practice specializes in diagnosing and treating ischemic heart disease and cardiomyopathies. His research contributed to the characterization of a novel condition known as "coronary microvascular dysfunction". He is a Fellow of the AHA, ACC, ESC and the Royal College of Physicians.

Section B — RESEARCH PROJECT DESCRIPTION

**Research Project Title**	**INTEGRATED STRATEGIES FOR THE STUDY OF TISSUE AND MOLECULAR DETERMINANTS OF VULNERABLE ATHEROSCLEROTIC PLAQUE**

**Keywords** (max. 5)	1. atherothrombosis
	2. coronary artery disease
	3. carotid stenosis
	4. inflammation
	5. immune response

**IRCCS INVOLVED**	*Number 17*
1.	IRCCS Centro Cardiologico Monzino
2.	IRCCS Fondazione Policlinico San Matteo
3.	IRCCS Istituti Clinici Scientifici Maugeri S.p.A. Società Benefit
4.	IRCCS Istituto Clinico Humanitas
5.	IRCCS Istituto di Ricerche Farmacologiche Mario Negri
6.	IRCCS San Raffaele Roma
7.	ISMETT Istituto Mediterraneo per i Trapianti e Terapie ad Alta Specializzazione
8.	IRCCS NEUROMED
9.	IRCCS Fondazione Istituto Neurologico Nazionale C. Mondino
10.	IRCCS Ospedale Policlinico San Martino
11.	IRCCS Ospedale San Raffaele
12.	IRCCS MultiMedica
13.	Fondazione Policlinico Universitario Agostino Gemelli IRCCS
14.	IRCCS Istituto Auxologico Italiano
15.	IRCCS Policlinico San Donato
16.	IRCCS SDN Synlab
17.	IRCCS Istituto Nazionale di Riposo e Cura per Anziani - INRCA

**Start Date**	**01.01.2022**
**End date**	**31.12.2022**
**Scientific project coordinator**	Prof. Giulio Pompilio<br>*Birth date:* October 1st 1964
affiliation<br>institution department/unit	**Centro Cardiologico Monzino, IRCCS**<br>Scientific Direction, Centro Cardiologico Monzino IRCCS
position title	Scientific Director and Head of Vascular Biology and Regenerative Medicine Unit at Centro Cardiologico Monzino IRCCS

telephone and e-mail	T +39 0258002456<br>giulio.pompilio@cardiologicomonzino.it

PERSONAL STATEMENT & short presentation of the Scientific project coordinator

Prof Giulio Pompilio graduated in Medicine and Surgery and specialized in Cardiac Surgery at the University of Milan with honours. He is Scientific Director and Director of the Unit of Vascular Biology and Regenerative Medicine at IRCCS Centro Cardiologico Monzino. He is currently chairman of the European Society of Cardiology Working Group CARE on cardiovascular regenerative and reparative medicine and Italian Representative of the EU Innovative Health Initiative. He was formerly an Italian Alternate Delegate at the advanced therapies committee (CAT) of EMA, Scientific Director of AriSLA and board member of Regione Lombardia Foundation for Biomedical Research.
The present project will focus on coronary and carotid atherosclerosis and related conditions with the overall goal to identify determinants of plaque vulnerability, integrating different research areas to address issues ranging from pathophysiology to clinical correlates. Within the Project, Prof. Pompilio will supervise the implementation efforts and the execution of planned activities to ensure in due time the achievement of the expected outcomes.

**TOTAL FINANCING REQUEST TO THE MOH**	*€ 1.728.020,00*	
Other Sources of Funds	*source of funding*	*(€)*

**OVERALL SUMMARY**	Atherothrombosis is a systemic disease caused by exogenous/endogenous vascular risk factors, affecting both coronary arteries and various other arterial districts. Coronary heart disease, for instance, is a dreadful expression of atherothrombosis, as almost 30% of first events are fatal. Being largely preventable, accurate atherosclerotic disease prediction may be lifesaving.<br><br>It is therefore clear that the identification of disease-specific factors that predispose to the onset, progression and/or instability of atherosclerotic lesions is of paramount importance. Their determination at the individual level would ultimately enable the design of new personalised therapeutic strategies, identifying actionable pathways and gene variants and developing molecules to activate protection and/or repair pathways.<br><br>This project was designed to investigate the tissue, cellular and molecular determinants of atherosclerotic plaque vulnerability, embracing a wide range of complementary and integrated fields of investigation. Three research areas were selected because they address crucial unmet needs and are particularly innovative: (*a*) atherosclerosis endophenotypes classified through complex diagnostic, prognostic or risk stratification biomarkers combining intravascular or non-invasive imaging data with genome-wide multiomics data; (*b*) impact of epigenetic factors on the progression and outcome of the disease; (*c*) immunological and/or neuroimmunological components associated with plaque instability. To this end, we will study several arterial districts, especially the carotid and coronary arteries, given existing clinical associations but also local differences in pathophysiology between them.<br><br>The project will leverage existing cases and biobanks (tissues, cells, sera or plasmas) with clinical follow-up, building on the work supported in the previous Network Project (Plaque Project, RCR-2018-2029). The methods envisaged range from diagnostic imaging examinations to multiomics

	molecular investigations.<br>With this project, the Cardiology Network intends to address major unmet needs, such as the definition of reliable biomarkers, the elucidation of molecular pathways and the identification of key cellular players for the implementation of better primary and secondary prevention strategies for atherosclerotic cardiovascular diseases. The project will fill the knowledge gap on atherothrombotic diseases by articulating investigations in different research areas, ranging from pathophysiology to clinical correlates of atherosclerotic plaque.
**STATE OF THE ART**	Atherothrombosis is a systemic disease caused by exogenous/endogenous vascular risk factors (VRFs), affecting both coronary arteries and various other arterial districts. Proposed cardiovascular disease (CVD) risk estimation tools, based on algorithms that weight the deleterious effect of VRFs, do not consider inter-individual susceptibility to VRFs or other protective/susceptibility factors and perform sub-optimally. Estimation systems are derived from cohort studies and risk estimates apply more to populations than to individuals. Therefore, there is a compelling need to improve individual risk estimates for more personalised and cost-effective prevention. Detection of carotid or lower-limb artery disease incorporates VRF exposure and individual susceptibility, improving the prediction of coronary artery disease (CAD). However, atherothrombosis in these districts typically develops later than in coronary arteries, limiting their incremental predictive value of CAD.<br>In this context, the identification of disease-specific factors that predispose to the initiation, progression and/or instability of atherosclerotic lesions is of paramount importance. Their determination at the individual level would make it possible to design new personalised treatment strategies by identifying actionable pathways and gene variants and developing molecules that activate protection and/or repair pathways. Genome-wide molecular profiling represents a promising tool to address this issue (Joehanes 2012): it allows early identification of 'pathognomonic' tissue and/or circulating biomarkers for timely diagnosis, risk stratification and early treatment options. This includes the characterisation of epigenetic factors, which provide a mechanistic link between environmental exposures and gene expression leading to disease (Khyzha 2017), and immune components, which have been shown to have a major role in the mechanisms of CVD and atherosclerosis progression (Ridker 2017).
**IMPACTS**	Despite enormous progress in diagnosis and therapy and reduction in associated mortality, atherosclerotic cardiovascular disease (ASCVD) is still the main cause of mortality in the world. Among the unmet needs, of paramount importance is the definition of reliable biomarkers, elucidation of molecular pathways and identification of key cellular players for the implementation of better strategies for primary and secondary prevention of ASCVD. With the present project, Rete Cardiologica intends to respond to the lack of knowledge about atherothrombotic diseases by articulating investigations in different research areas, ranging from pathophysiology to clinical correlates of atherosclerotic plaque, building on studies already undertaken by the Rete itself.<br>Over the past decades, enormous efforts have been devoted to the identification of new biomarkers that can complement traditional VRFs in the reclassification of cardiovascular (CV) risk. This project, which

	provides novel biomarkers to be used in addition to other VRFs already available, as well as advanced pathophysiological knowledge, has a high potential to improve the current ability to assess CV risk. Furthermore, the results of this project may pave the way for substantial advances in the management of ASCVD patients, helping to build a framework for precision medicine applications. Finally, the development and validation of new individual tissue and/or circulating signatures will allow earlier and better recognition of subjects at higher risk of ischaemic CV diseases. This could lead to a further reduction in CV events and hospitalisation, including sequelae and late complications such as heart failure, which represent a major cost to the National Health Service.
**EXPECTED RESULTS**	The added value of a Network project lies in the possibility of interaction of different expertise, exchange of data and samples, broader access to case studies, and collaboration on specific topics. Substantial added value resides in the multiplier effect of integrating data from different sources by focalising the response on unmet needs and being able to perform systems medicine approaches. In this project, we can thus study the determinants of atherosclerotic plaque in different arterial districts (carotid, coronary, femoral) and most novel components (omics and imaging biomarkers, epigenetic factors, immune responses).<br>General expected results are:<br>✓ A spatial transcriptomic map of human carotid atherosclerotic plaques<br>✓ A set of tissue or circulating biomarkers (circRNAs, inflammatory factors mediators of calcification) of carotid plaque vulnerability<br>✓ A high-intensity exercise programme for carotid stenosis reduction<br>✓ Two models of coronary plaque neoangiogenesis<br>✓ Endophenotypes of coronary plaque instability in acute coronary syndromes (ACS) combining intravascular imaging and molecular biomarkers<br>✓ Implementing the best diagnostic computed tomography (CT) based pathway for patients with CAD at intermediate-high risk<br>✓ Distinctive multiomic and CT integrated signatures of endophenotypes of CAD burden, progression and outcomes<br>✓ Cellular and/or circulating epigenetic factors (microRNAs, non-coding RNAs, DNA methylation, and variations in chromatin accessibility) involved in the pathogenesis or associated with different outcomes of carotid and coronary atherosclerosis<br>✓ Clarify the role of circulating or infiltrating NK cell subsets in carotid atherosclerosis progression<br>✓ Characterization of cellular components of the neuro/immune response (NK subsets, infiltrating immune cells, autophagy, sympathetic or parasympathetic innervation) in carotid plaque vulnerability<br>✓ Role of the initiator of the lectin pathway of the complement system in carotid plaques.
**GENERAL AIM**	The overall aim of the project is to gain insights into the tissue, cellular, circulating and molecular determinants of atherosclerotic plaque vulnerability. In this respect, we propose a wide range of complementary and integrated fields of investigation. In particular, three fields have been selected because they respond to unmet needs and because they are particularly innovative: (*a*) the search for complex diagnostic, prognostic or risk stratification biomarkers, *i.e.* those that combine intravascular or non-invasive imaging data with genome-wide multiomics data; (*b*) the study of the impact of epigenetic factors on both the progression and the

	outcome of the disease; (*c*) the characterisation of immunological and/or neuroimmunological components associated with plaque instability. Given the clinical associations but also the local differences in pathophysiology between them, we will study several arterial districts, especially the carotid and coronary arteries.	
**SPECIFIC OBJECTIVES & HYPOTHESIS**		
**Specific objective 1 – WP1.1**	**Indicator**	**Expected value**
Transcriptomic atlas generation of human atherosclerotic plaques at the spatial-single-cell level	1. Sample preparation	Sufficient material for transcriptional analysis
	2. Sample analysis, including bioinformatics	Construction of spatial transcriptomics maps
	3. In vitro biological function testing of the identified genes	Successful gene silencing
**Specific objective 2 – WP1.2**		
To evaluate circular RNAs and inflammatory-cytokine signatures in carotid plaques and peripheral blood of patients undergoing carotid endarterectomy, correlating the results with plaque vulnerability	1. Measurement of atherosclerosis-related circRNAs	Deregulation in patients with vulnerable plaque compared to patients with stable plaque
	2. Measurement of inflammatory cytokines	Higher in patients with vulnerable plaque compared to patients with stable plaque
**Specific objective 3 – WP1.3**		
To evaluate the pathophysiological and prognostic role of inflammatory and calcification biomarkers on plaque vulnerability in patients with severe carotid stenosis undergoing endarterectomy	1. Number of samples assessed for FGF23 levels	286
	2. Number of samples assessed for Klotho levels	286
	3. Number of samples assessed for JCAD levels	286
	4. Number of samples assessed for ZAG levels	286
**Specific objective 4 – WP1.4**		
Evaluation of the impact of (*i*) 6-hour/week intensity resistance exercise programme on the size of carotid plaque and (*ii*) high-intensity training program on functional capacity and quality of life	1. Carotid plaque extension (expressed in mm)	15% reduction in plaque size
	2. Amount of calcium inside the plaque	20% increase in intraplaque calcium
	3. Six minutes walking test	Significant at α 0.01
	4. Kansas City Cardiomyopathy Questionnaire (KCCQ)	Significant at α 0.05
**Specific objective 5 – WP1.5**		
Identification of the immuno-phenotypic and morpho-functional bases for the distinction of the two mechanisms of plaque neoangiogenesis: the atherosclerotic and the thrombotic/thromboembolic models	1. Immunohistochemical and morpho-structural studies on thrombo-aspirated material from ACS patients and thromboendarterectomy samples obtained from patients with CTEPH	Definition of the immune-morpho-functional features of plaque neoangiogenesis
**Specific objective 6 – WP1.6**		
To establish an efficient and cost-effective biomarker panel for identifying homogeneous ACS patient subsets according to Optical Coherence Tomography (OCT) interrogation of the culprit plaque	1. Enrolment status	Planned patients and controls (160 ACS, 50 SA, 50 HC)
	2. OCT analysis of ACS enrolled patients	Identification of a homogeneous subset-of ACS according to plaque morphology

	3. Biomarker identification	Identification of specific biomarkers associated with plaque morphology
**Specific objective 7 – WP1.7**		
To evaluate the 1-2-year prognosis of CAD patients undergoing standard-of-care *vs.* cardiac CT + CT perfusion and correlate this with high-risk plaque features, myocardial ischemia and blood mRNAs	1. Enrolment of patients with stable chest pain	55 patients enrolled
	2. Acquisition of cardiac CT and CT perfusion images	Whole cohort image dataset
	3. Collection of blood samples for RNA isolation	RNA with RIN >8
**Specific objective 8 – WP1.8**		
To identify combined omics and imaging biomarkers predicting CAD progression and/or incident events at medium-term follow-up	1. Baseline and follow-up coronary computed tomography angiography (CCTA) analysis	140 patients
	2. Baseline blood methylome and transcriptome data combined with genome-wide genotypes	Prediction of baseline and/or follow-up CCTA features
**Specific objective 9 – WP2.1**		
Evaluation of non-coding RNA expression as new biomarkers of atherosclerosis in obstructive sleep apnoea (OSA) and their correlation to epigenetic age after continuous positive airway pressure (CPAP)	1. Analysis of miR-485-5p on a new sample of patients	Down-regulated in OSA patients with/without carotid plaque. Restored after CPAP
	2. Investigation of MALAT1and BACE-1AS in at least 100 patients	Modulation of MALAT1and BACE-1AS in OSA with/without plaque. Restored after CPAP
	3. Investigation of epigenetic age in at least 100 patients	Differentially modulated in pathological conditions compared to controls. Restored after CPAP
**Specific objective 10 – WP2.2**		
Proof of concept on the association between abdominal aortic aneurysm risk and circulating P2X purinoceptor 7 and/or its targets miR-150 and miR-186 in patients with carotid atherosclerosis	1. Levels of circulating P2X purinoceptor 7 in patients and matched controls	Identification of patients at higher risk of developing aortic aneurysm
	2. Levels of circulating miR-150 and miR-186 in patients and matched controls	Identification of patients at higher risk of developing aortic aneurysm
**Specific objective 11 – WP2.3**		
To identify correlations between molecular, epigenetic, and gene profiles, extent and severity of CAD, patient demographic and clinical data, for personalized management of individual subjects	1. Identification of the dysregulated epigenetic markers	Quantification of significant differentially accessible regions (DARs) and related differentially expressed genes (DEGs) in patients with various degrees

		of CAD severity
	2. *In vitro* validation	Real-time PCR expression evaluation of top-ranked DEGs
	3. Clinical associations and correlation with imaging parameters	Significant correlations of dysregulated gene expression with demographic and. clinical information and CAD severity
**Specific objective 12 – WP2.4**		
Identification of miRNAs associated with coronary atherosclerosis, both in coronary and venous blood and in cells of stable and unstable patients; estimation of acute coronary event risk	1. Protocol approved by Ethical Committee	Month 1
	2. Pilot study	Ten patients
**Specific objective 13 – WP2.5**		
To identify molecular signatures of exosomal microRNAs in patients at risk for atherosclerotic cardiovascular disease	1. Biobanking of biological samples of patients at risk of atherosclerotic cardiovascular disease	Valid samples from 140 CAD patients
	2. Screening of exosomal microRNA profile of patients enrolled	At least 140-150 measurable microRNAs
	3. Clinical data collection to identify a microRNA signature of atherosclerotic cardiovascular disease	A panel of 5-10 top differentially expressed microRNAs
**Specific objective 14 – WP3.1**		
Profiling Natural Killer (NK) cell subsets (circulating and plaque infiltrating) in peripheral artery atherosclerotic diseases and investigating the functional crosstalk between them and plaque cellular components	1. NK immunophenotyping	Detection of membrane antigens related to maturation and activation of NK cells
	2. Co-culture of NK cells and CD14+ derived macrophages	Influences on macrophage polarization
**Specific objective 15 – WP3.2**		
Analyse the immune cells infiltrating and aggregating in atherosclerotic plaques to evaluate the contribution and localization of specific cell subpopulations in the arterial wall remodelling process	1. Characterization of immune cells infiltrating atherosclerotic plaque	An increased presence of infiltrating immune cells in atherosclerotic plaque.
	2. Characterization of the autophagic profile of immune cells	Dysregulation of the expression levels of the autophagic markers
	3. Autonomic nervous system innervation	Increased density of NF200+ and TH+ axons in the plaques
**Specific objective 16 – WP3.3**		
Characterization of intraplaque and circulating ficolin-2 molecular fingerprints (oligomerization and binding with the serine proteases MASPs)	1. Non-reducing Western blot-calculated molecular weight	kDa of protein molecular weight will indicate oligomerization of ficolin-2 in plaque

		with different morphologies
	2. Immunoprecipitated signal	The optical density of the co-immunoprecipitated signal should reveal ficolin-2-MASPs interaction
	3. Molecular changes of circulating ficolin-2 before and after endarterectomy	kDa of protein molecular weight will indicate oligomerization of ficolin-2 in blood obtained from the same patient at different time points from surgery

**Milestones of the project**	*Milestones*	*Month*
	1. Kick-off Meeting	**1**
	2. Report on interim analyses	**6**
	3. Final Report	**12**

**Major Activities**	The project is divided into three WPs, focusing on three types of activities.<br>• Search for tissue and circulating biomarkers.<br>○ Spatial transcriptomics, circular RNAs, inflammatory cytokines, calcification-mediating molecules, and high-intensity exercise programme in patients with ***carotid atherosclerotic disease***<br>○ Neoangiogenesis, molecular mechanisms of coronary instability, stress cardiac CT myocardial perfusion, CCTA, blood multiomics in patients with ***coronary atherosclerosis***<br>• Search for epigenetic factors.<br>○ OSA, DNA methylation age, microRNAs and ***carotid atherosclerosis***<br>○ Chromatin accessibility, circulating (and exosomal) and cellular microRNAs in ***coronary atherosclerosis***<br>• Immunological features in the pathogenesis of atherosclerosis.<br>○ NK cells, neuroimmune factors, autophagy, and the complement system in ***carotid atherosclerosis***.
**Methods**	The general methodological approach adopted is, where possible, to exploit existing cases and biobanks of tissues, cells, sera or plasmas with follow-up, building on the work already supported in the previous Network Project (Plaque Project, RCR-2018-2029). Where individual tasks require it, new patients will be enrolled.<br>The methods envisaged range from diagnostic imaging examinations to molecular investigations. In particular, we will employ instrumental investigations of invasive intravascular (OCT) and non-invasive imaging (echo-colour-doppler, CCTA, stress CTP, MRI) coupled with the collection of anamnestic, semeiotic and laboratory data for the precise definition of atherosclerotic disease phenotypes. The wide range of

	molecular tests includes next-generation sequencing (NGS) technologies, microarrays, quantitative PCR, digital PCR, immunoassays, Western blot, and immunohistochemistry. Each WP is divided into a number of tasks, each detailing the methodological approaches adopted.

PROJECT IMPLEMENTATION

**WP 1**	**TISSUE AND CIRCULATING BIOMARKERS**
IRCCS WP leader	Centro Cardiologico Monzino, IRCCS
WP 1 description	*Aims*<br>**Task 1.1.** (*i*) To generate a comprehensive atlas of gene expression of human carotid atherosclerotic plaques at the spatial level. (*ii*) To test whether the modulation of specific genes previously identified regulates smooth muscle cells *in vitro*.<br>**Task 1.2.** To establish a possible correlation between plaque vulnerability and the expression of atherosclerosis-related circRNAs and/or inflammatory cytokines in carotid plaques and peripheral blood of patients undergoing carotid endarterectomy.<br>**Task 1.3.** To evaluate the association and prognostic role of potential calcification biomarkers (FGF23/Klotho, JCAD, and ZAG) on plaque vulnerability in samples from 286 patients with severe carotid stenosis undergoing endarterectomy.<br>**Task 1.4.** To assess the impact of a high-intensity resistance exercise programme on carotid plaque size.<br>**Task 1.5.** (*i*) To identify neoangiogenesis in thrombo-aspirated material retrieved *in vivo* from patients with acute coronary syndromes (ACS) who underwent percutaneous coronary intervention (PCI) preceded by intracoronary imaging. (*ii*) To correlate neoangiogenesis with markers of previous haemorrhagic plaque events (glycophorin A and iron deposits) and healing/reabsorption processes of thrombotic material. (*iii*) To establish the relationship between the plaque substrate of neoangiogenesis (pultaceous-lipid core, fibrous cap, inflammation, and calcifications) and plaque haemorrhage and neoangiogenesis. (*iv*) To define 2 models of arterial neoangiogenesis: *(a)* the atherosclerotic model (*i*-*iii*) and *(b)* the pure thrombotic model in chronic thromboembolic pulmonary hypertension (CTEPH), where neoangiogenesis is exclusively sustained by the healing of thromboembolic material (*iv*).<br>**Task 1.6.** (*i*) To identify homogeneous subsets of ACS patients at optical coherence tomography (OCT) interrogation of the culprit plaque. (*ii*) To assess the role of shear stress in coronary instability through OCT-based reconstruction of coronary lesions and fluid dynamics studies. (*iii*) To establish the most efficient and cost-effective biomarker panel for the identification of homogeneous ACS patient subsets. (*iv*) To unravel specific mechanisms and players of coronary instability and to identify molecular therapeutic targets, taking advantage of omics technologies. (*v*) To design animal models testing new molecular pathways and therapeutic strategies.<br>**Task 1.7.** (*i*) To evaluate the impact of computed tomography myocardial perfusion (CTP) and coronary computed tomography angiography (CCTA) *vs.* standard-of-care functional assessment on the prediction of major adverse cardiovascular events (MACE) in patients with nonobstructive or obstructive CAD. (*ii*) To compare the impact on the reclassification rate of CCTA due to the addition of CTP. (*iii*) To identify blood-based transcriptional biomarkers of CAD subtypes.<br>**Task 1.8.** (*i*) To identify associations between individual blood multiomics profile and severity and/or high-risk features of coronary atherosclerosis in patients with suspected CAD and (*ii*) to seek for combined omics and imaging biomarkers predicting disease progression and/or incident events at medium-term follow-up.
	start date-end date: **01/01/2022 – 31/12/2022**
	Activities of the WP<br><br>**Task 1.1.** ***Spatial transcriptomics of carotid plaques.***<br>Our understanding of the cellular heterogeneity and tissue architecture of human carotid atherosclerotic

plaques has been largely derived from histology, bulk sequencing, low dimensionality hypothesis-based studies, and experimental model systems (Tomey 2014). Single-cell RNA sequencing (scRNAseq) has offered remarkable new opportunities to systematically describe the cellular landscape of plaques and reveal new insights into cell biology, disease pathogenesis, and drug response. Several studies have successfully applied scRNAseq to selected populations in mouse plaques, such as vascular smooth muscle cells (SMCs), to reveal a continuum of differentiation states within specific cells (Wirka 2019; Pan 2020). Therefore, a more detailed transcriptional atlas of human plaques at high molecular resolution, representative of all cell subtypes, is required to further define the taxonomy of the disease, identify heterotypic cellular interactions, and determine cellular differentiation events, in particular, induced by diabetes. Just as importantly, data systematically mapping the spatial transcriptomic architecture of atherosclerotic plaques, which can determine how cells in the tissue are organized as functional units, are absent.
Activities include (*a*) generation of spatial transcriptomic atlas of human carotid atherosclerotic plaques at the single-cell level and generation of vessel transcriptomic data; (*b*) *in vitro* modulation of top genes (identified in previously performed experiments, cf. the Scientific Report of Ricerca Corrente Reti-RCR 2019) in atherosclerotic plaque SMCs of HFD ApoE-/- mice using the siRNA technology.
Our research will help to obtain essential information on how this complex cellular communication works in atherosclerotic plaques, with particular attention to the influence of diabetes on such pathological processes. We plan to generate a comprehensive transcriptomic database of human atherosclerotic plaques that can be then utilized for different purposes. We are confident we to be able to identify new cell populations with a specific gene expression profile in human atherosclerotic plaques

Task 1.2. ***Circular RNAs and inflammatory cytokines in carotid plaque vulnerability.***
Enrolled patients (n= 50) will undergo carotid endarterectomy according to the usual clinical practice. Upon entering the operating room, 4 ml of venous blood will be collected in 6 tubes with EDTA anticoagulant, from peripheral access. Peripheral blood mononuclear cells (PBMC) will be separated and isolated from the venous blood sample by the Percoll gradient. The expression of atherosclerosis-related circRNAs will be evaluated on PBMC by qRT-PCR. On the serum of the same samples, the quantitative expression of cytokines involved in inflammation and the atherosclerotic process (including IL-6, IL-1β, TNF-α, and IFN-γ) will be evaluated using the ELISA method. Negative controls will be constituted by age and sex-matched healthy controls (n=15). Atherosclerotic plaque samples will be harvested and plaque vulnerability will be determined by histology analysis. The levels of atherosclerosis-related circRNAs will be quantified by digital PCR.
Three Units of the IRCCS Policlinico San Donato will be involved in this task. The Vascular Surgery Unit is a national referral centre for carotid disease, with internationally recognized expertise. Annually about 50-60 carotid endarterectomy procedures are performed. The Vascular Surgery Unit therefore will perform patients' enrolment with the collection of blood and vascular samples, that will be stored at the institutional Biobank (BioCor). The Pathology Unit will be in charge of the histologic analysis to evaluate plaque vulnerability. The Molecular Cardiology Laboratory has a long-time experience in studying noncoding RNA involvement in cardiovascular disease, taking advantage of the most advanced technologies in the field of molecular biology.
We expect patients with a vulnerable carotid plaque to have higher levels of circulating pro-atherosclerotic cytokines and that the same cytokines will be more expressed in vulnerable plaques when compared to stable carotid plaques. We also expect to identify atherosclerotic-related circRNAs deregulated in PBMC plaques of patients with vulnerable plaques and, therefore, to better define the molecular pathways involved in the pathogenesis of plaque destabilization, to define possible preoperative diagnostic biomarkers of plaque vulnerability from a personalized medicine perspective.

Task 1.3. ***Pathophysiological and prognostic role of inflammatory and calcification-mediating molecules in carotid stenosis.***
Among the various mechanisms involved in the rupture and/or erosion of atherosclerotic plaque, inflammation and calcification phenomena are becoming increasingly important. More specifically, both the receptor activator of nuclear factor-κB ligand (RANKL) and osteopontin (OPN) appear to be deeply involved in the recruitment and activation of neutrophils within the atherosclerotic plaque. In keeping with

previous projects (RCR 2019), we propose to continue the study of this complex signalling network within the cohort of patients with severe carotid stenosis undergoing endarterectomy. This cohort counts 286 cases (from which serum and plaque histological samples are available). The main activity of this RC2022 is finalizing the study of the FGF23/Klotho axis as well as introducing novel biomarkers, i.e. junctional protein associated with coronary disease (JCAD) and Zinc-α2-glycoprotein (ZAG). Traditionally implicated in the homeostasis of circulating phosphate levels, the FGF23/Klotho axis is involved in vascular calcification processes, but its role in atherosclerosis and possible pro-inflammatory activity remain largely unexplored. Preliminary data by the IRCCS San Martino research group are encouraging and suggest a significant pathophysiological role of bone metabolism mediators in atherosclerotic disease. JCAD, a membrane protein whose function remained unknown for years, has been recently associated with an increased risk of atherosclerosis by genome-wide association studies (GWAS). Mechanistically, JCAD plays a role in endothelial inflammation and arterial thrombosis, a dreadful but common sequela of atherosclerotic cardiovascular disease that can result in life-long disability, heart failure, and eventually premature death. Preliminary results implicate JCAD plasma levels in CAD severity and thus might be a promising tool to guide secondary prevention in patients at high risk for adverse events. Similarly, ZAG is a newly identified adipokine that is associated with lipid metabolism, vascular inflammation and fibrosis. In keeping with its role as a negative modulator of inflammation, ZAG levels are lower in patients with coronary heart disease than in healthy controls. Yet, evidence on its association with atherosclerosis remains scarce and to date its prognostic role in such patients is unexplored.

Previous findings (RCR 2019) unveiled the role of OPN as a predictor of adverse outcomes. Specifically, serum OPN levels correlated positively with the expression of inflammatory mediators within the carotid plaque and predicted major cardiovascular events two years after endarterectomy (Carbone 2018). Besides, we identified the platelet-to-lymphocyte ratio as a predictor of acute coronary syndrome at an 18-month follow-up in the same cohort (Bonaventura 2019). Current RC 2022 aims at completing the assessment of the FGF23/Klotho axis and continuing the enrolment of novel 10 patients for assessing biomarker stability over time. Further, it expands the analysis to the novel biomarker JCAD and ZAG. The study will be conducted by Prof. Fabrizio Montecucco (PI), in collaboration with Prof. Luca Liberale, Prof. Federico Carbone (Clinica di Medicina Interna 1) and Prof. Pratesi (Unità di Chirurgia Vascolare ed Endovascolare).

Aimed at clarifying the role of the inflammatory-calcification axis in the pathophysiology and prognosis of carotid atherosclerosis, the following activities are set:

1. Quantification of the FGF23/Klotho axis, JCAD, and ZAG at baseline in serum samples of patients with carotid atherosclerosis by commercially available ELISA kits.
2. Long-term follow-up (up to 18 months) to evaluate, retrospectively, the prognostic role of the FGF23/Klotho axis, JCAD and ZAG in the already recruited cohort of patients with carotid stenosis undergoing endarterectomy (n=286). Relevant clinical events such as death from cardiovascular causes, ischaemic stroke and myocardial infarction will be included as outcomes.
3. Enrolment of new patients to validate stability during follow up of plaque calcification biomarkers (blood sampling at enrolment and one-year follow-up). We estimate to enrol additional 10 patients with severe carotid stenosis undergoing endarterectomy.

Statistical Analysis. A largely validated cohort of 286 patients with severe carotid atherosclerosis is already available (Montecucco 2010 and 2011). For the longitudinal cohort study, the sample size was calculated considering a 4-fold increase in the incidence of major cardiovascular adverse events, as already observed in previous studies (Montecucco 2011, Dimitrijevic 2006, Gordin 2014, Kanbay 2014). Based on the power calculation for the log-rank test, the minimum sample size required to detect a 4-fold increase in the risk of adverse cardiovascular events (2% vs. 8%) with a power of 80% and an α-error of 5% was 185 patients.

Mediators of inflammation-calcification interaction might play a role in determining plaque vulnerability and, therefore, become novel potential therapeutic targets in carotid atherosclerosis. Moreover, this task might open the way to a multidisciplinary approach (beyond interventional only) to patients with advanced atherosclerosis.

Task 1.4. *High-intensity exercise programme in patients with atherosclerosis carotid disease.*

The results obtained in the "Integrated Strategies for the Study of Cellular and Genomic Determinants of the Atheromatous Plaque" sub-project devoted to exercise training, using combined aerobic and resistance

methods as innovative tools, have shown an improvement in functional capacity and quality of life in the patients enrolled. However, there was no detectable reduction in the size of both carotid and coronary plaques, respectively evaluated by carotid Doppler and MRI.

A literature review in this regard shows how these experiences made in the field indicate that while high workloads are necessary to halt the progression of coronary atherosclerotic lesions (1,533 ± 122 kcal/week), regression of coronary lesions is observed only in patients expending an average of 2,200 kcal/week in leisure-time physical activity, amounting to ~ 5 to 6 h/week of regular physical exercise (Hambrecth 2010).

In light of existing literature, an evaluation of the efficacy of a high-intensity exercise programme in patients known for carotid disease (with carotid plaque >70%) was hypothesized. The training programme is exclusively based on resistance exercises involving sessions of about 6 hours per week with an energy expenditure of at least 2500 Kcal/week.

Ten patients (who participated in the previous carotid plaque sub-study with the double advantage of a short enrolment period and fair compliance to training programs) will be enrolled within two months from the start of the project. For six months, they will perform a high-intensity training program as described above, to evaluate the impact of exercise training on the size of the carotid plaque. Patients will be evaluated with carotid Echo-Colour-Doppler. The impact of this program on functional capacity and Quality of Life will be assessed by the six-minute walking test (6MWT) and the Kansas City Cardiomyopathy Questionnaire (KCCQ).

The expected results of this activity are to improve or halt the progression of atherosclerotic lesions and ameliorate the quality of life through resistance exercise. The impact and evaluation of a high-volume resistance exercise programme on carotid plaque size and composition will allow individually tailored exercise programmes in subjects with carotid artery disease.

Task 1.5. ***Neoangiogenesis in atherosclerotic coronary plaques causing acute ischemic syndromes.***

Neoangiogenesis is a common reparative process in atherosclerotic, atherothrombotic, and thromboembolic diseases. Its presence is both an indicator of cytokine-induced pro-neoangiogenic stimuli (and therefore it is mediated by inflammation) and evidence of activation of mechanisms of repair/reabsorption of haemorrhagic extravasations, with the organization of thrombotic-haemorrhagic material (PMID: 12117850; 22828775; 21398643; 30414747; 27052269; 12117850; 35449407).

In this task, the IRCCS San Matteo will selectively explore the presence and morpho-structural features of neoangiogenesis in thrombo-aspiration material retrieved *in vivo* from patients with ACS treated with PCI and preceded by coronary intravascular imaging (OCT) (*in vivo* atherosclerotic model) *vs.* that observed in the spontaneous thrombotic-only model of CTEPH.

Planned activities are:

1. *In situ* immunohistochemical search for the expression of cellular markers of neoangiogenesis.
2. Morpho-structural characterization of neoangiogenic cells (single cells, aggregated clusters, capillaries, or other microvascular structures).
3. Definition of relationships between neoangiogenesis and thrombosis in the context of the thrombosed material (glycophorin-A-positive material, intra-, and extra-cellular iron deposits).
4. Comparison of the immuno-morpho-functional characteristics of neoangiogenesis in the atherosclerotic context with the purely and primarily thrombotic model observed in CTEPH.

To this end, San Matteo will perform immunohistochemical and morpho-structural studies on thrombo-aspirated material from ACS patients and thromboendarterectomy samples obtained from patients with CTEPH, to identify the immuno-phenotypic and morpho-functional bases for distinguishing two models of coronary plaque neoangiogenesis: (*a*) the atherosclerotic model; (*b*) the thrombotic/thromboembolic model. Indeed, we foresee two main mechanisms of plaque neoangiogenesis: (*a*) type 1, induced by cytokine stimuli released by the plaque inflammatory cells in the thrombo-aspiration material from patients with ACS; (*b*) type 2, induced by the thrombotic material and associated with thrombus reabsorption and organization in the thromboendarterectomy samples obtained from patients with CTEPH undergoing surgery. This information can provide the basis for defining the contribution of haemorrhagic events in the progression of atherosclerotic plaques.

Task 1.6. ***Identification of molecular mechanisms of coronary instability in homogeneous subsets of***

patients with acute coronary syndromes.

This Task will be developed into 4 subtasks.

1.6.1. Patient recruitment and clinical evaluation, biological samples collection and storage, coronary imaging acquisition by OCT and analysis. We will prospectively enrol: 1) patients with an admission diagnosis of non-ST-segment elevation myocardial infarction (NSTEMI) according to current European guidelines; 2) consecutive patients with a diagnosis of stable angina (SA) according to current European guidelines. NSTEMI (within 12 hours of symptom onset) and SA patients will be enrolled at the time of their admission to the Coronary Care Unit and Cardiovascular Departments; (3) in the same period, consecutive healthy controls (HC), age and sex-matched 1:3 with NSTEMI patients, will be enrolled at the outpatient clinic for cardiovascular risk factors control.

Biological sample collection and storage. In NSTEMI patients, venous blood samples will be taken at the time of patient enrolment, within 12 hours from symptom onset and before the interventional procedure. Coded plasma, serum samples and cell pellets will be stored at -80°C. Faecal samples will be obtained as soon as possible and frozen at -80°C immediately after collection. In NSTEMI patients, at the time of coronary angiography and before PCI procedures are performed (within 48 hours from patient enrolment), frequency-domain OCT images will be acquired by a commercially available system connected to an OCT catheter. OCT image analysis will be performed offline by 2 expert investigators blinded to all the other findings; discordance will be resolved by consensus. Culprit lesion morphology will be described according to previously reported criteria. OCT images will be acquired also in SA patients. NSTEMI patients presenting a smooth culprit plaque will undergo intracoronary acetyl-choline testing.

At the time of coronary angiography, when a macroscopic thrombus can be identified in the culprit coronary lesions, NSTEMI patients will undergo thrombus aspiration. Thrombus aspirates will be used either to obtain lysates or they will be included in paraffin to perform immune-histochemical analysis.

1.6.2. OCT-based reconstruction of coronary atherosclerosis and fluid dynamic study to assess the role of shear stress. Diagnostic coronary angiography, together with OCT imaging, will be used to perform 3D reconstruction of coronary arteries through quantitative coronary analysis (QCA). Computational fluid dynamics techniques will be used to simulate pulsatile blood flow through the reconstructed patient-specific coronary artery models by solving Navier-Stokes equations. Indices of Wall Shear Stress (WSS), in particular time-averaged WSS and oscillatory shear index (OSI), will be computed.

1.6.3. Identification of specific biomarkers and molecular pathways in NSTEMI patients for predicting OCT phenotypes.

Biomarkers of Plaque Rupture with systemic inflammation. We will assess markers of innate and adaptive immunity by multiplex cytokine panels including biomarkers of Th1-dependent immune response, Treg response, autoimmunity and innate immunity response. The role of altered glucose metabolism in T-cell dysregulation will be investigated. To this end cell-surface expression of the glucose transporter type 1 (GLUT-1), the glycolysis related factor PKM2 and the glucose internalization will be assessed by flow cytometry on CD4+ T-cells. Thrombus aspirates will be analysed to assess the frequency of the different T-cell subsets and the expression of the immunomodulatory molecule CD31.

Biomarkers of Plaque Rupture without systemic inflammation. In all enrolled patients, in vivo platelet activation will be evaluated with a broad panel of soluble markers, including serum thromboxane (TxB2, the stable metabolite of TXA2) and the products of receptor shedding (sGPIb, glycocalicin), sGPV, sGPVI, sP-selectin, sCD40L), which will be measured by ELISA. Western blot analysis will also be employed to evaluate whether platelets have a more immune/inflammatory or aggregation-prone phenotype, based on the levels of established protein markers, e.g. TLRs *vs.* integrin IIb3. We will investigate the role of the major activator of the integrin β, the small GTPase-Rap1, and its regulators CalDAG-GEFI and RASA3. Levels of oxidative stress markers will be measured, such as soluble NOX2 derived peptide (sNOX2dp), plasma urinary 8-iso-PGF2 (Oxiselect 8-iso-PGF2 ELISA kit), and ox-LDL. Platelet activation and oxidative stress markers will be also assessed in thrombus aspirates.

Biomarkers of Plaque Erosion. Markers involved in shear stress, hyaluronan metabolism and neutrophil activation will be assessed. In detail, *ex vivo* and *in vitro* experiments will be developed to evaluate the role of shear stress exposure. To this end, primary human aortic endothelial cells (HAOECs), cryopreserved and derived from a single donor, will be incubated with serum and/or with PBMCs isolated from NSTEMI patients with documented altered WSS. Haemodynamic alterations will be also reproduced on HAOECs

exposed to mechanical stress and controlled flow variations using the Ibidi Pump system. After *ex vivo* and *in vitro*, mRNA expression levels of HYAL2, CD44 splicing variant 6 and TLR2 will be assessed in HAOEC by qPCR, while IL8, IL1, nitric oxide (NO) will be measured in culture supernatants by ELISA kits. To test neutrophil activation, myeloperoxidase (MPO), Cathepsin G, circulating neutrophil lipocalin (NGAL), and circulating markers of Neutrophil extracellular traps (NETs) will be measured by immune-enzymatic assays in patients' plasma. Finally, thrombus aspirates from NSTEMI patients will be analysed to characterise the presence of neutrophils and NETs, proteins involved in plaque erosion, such as CD44v6 and HYAL2, and markers of oxidative stress, such as MPO. Furthermore, to test the hypothesis of a microbial trigger in thrombus formation, bacterial LPS and its correlations with NETs will be also evaluated.

Biomarkers of Smooth Plaque. We will measure the levels of circulating catecholamines and hair cortisol (reflecting chronic stress in the 3 months before the event). Furthermore, Rho kinase activity will be measured in leukocytes.

1.6.4. Role of the microbiome in plaque instability.

Metagenomes of gut microbiota. The taxonomic diversity and the functional capacity of the gut microbiota will be assessed by 16S rRNA gene analysis and by integrating the relative gene abundance with metabolic networks, respectively. Furthermore, microbial-derived trimethylamine N-oxide (TMAO) metabolite will be assessed in serum.

Animal model to test the role of the microbiome in plaque instability. The role of the gut microbiome and TMAO microbial metabolite in pathways correlated with plaque phenotype will be proven in murine models of atherosclerosis. To this end, a murine model of human gut microbial transplantation will be developed. Stool samples from NSTEMI SA patients and HC will be transplanted in germ-free ApoE-/-mice subdivided into three groups each with a different diet (chow diet, choline diet and choline diet supplemented with an inhibitor of TMAO production (DMB). After 20 weeks, animals will be sacrificed and analysed for aortic root atherosclerosis area quantification, inflammatory cell content and systemic level of various biomarkers described in the subtask 1.6.3.

Different Units of the IRCCS Policlinico Universitario Agostino Gemelli will be involved in this task. The Department of Cardiovascular Science is equipped with a Coronary Care Unit with 14 monitored beds and a Sub-intensive Care Ward with 18 beds that admit at least 300 patients per year with a diagnosis of NSTEMI, a Cath Lab with two fully equipped operating rooms where percutaneous coronary interventions are performed h24. Cath Lab is equipped with a frequency domain OCT system (C7-XR or ILUMIEN OPTIS, St. Jude Medical, St. Paul, MN). The Cellular and Molecular Cardiology Lab is strategically located within the Cardiovascular Department. It is equipped with various instruments for cellular and molecular biology and offers specific expertise in these fields.

This task should advance the state of the art in the field of cardiovascular health promotion and secondary prevention of ACS. This proposal has been conceived with the primary mission of delivering new knowledge in the management of ACS, by the identification of molecular mechanisms of disease in homogeneous subsets of patients. The identification of homogenous groups of patients with a common mechanism of coronary instability identified by OCT imaging and, more importantly, by specific biomarkers, is the premise for the identification of more accurate diagnostic and prognostic algorithms and novel therapeutic targets and might pave the way for the implementation of Precision Medicine in this field. The integrated analysis of omics results will extract biologically meaningful information from wide datasets and will give us the possibility to test, in future projects, the role of specific environmental factors by functional *ex vivo* and *in vivo* models. The knowledge gained from research performed in this Task will lead to an improvement in the prevention and treatment of ACS, which are a major cause of ill health and death in Italy, Europe and worldwide.

Task 1.7. *Impact of stress cardiac CT myocardial perfusion on the prognosis of patients with CAD.*

This task will be focused on elucidating the impact on the short-term prognosis (1-2 years) of a diagnostic approach based on CCTA with or without the addition of perfusion imaging in patients with stable chest pain compared with the standard-of-care functional tests. Consecutive patients will be randomized to Group A (CCTA assessment) or Group B (standard-of-care functional tests). Group-A patients with obstructive CAD will be further evaluated with stress CTP. CCTA images will be analysed for stenosis extent and plaque characteristics.

Inclusion criteria are age >18 years and suspected or known CAD with new symptoms onset, referred for clinical indication to non-urgent diagnostic assessment. Exclusion criteria are non-invasive diagnostic testing within 90 days before enrolment; pre-test probability of obstructive CAD <15%; ACS; the need for an emergent procedure; contraindication to the administration of contrast agent, sublingual nitrates, β-blockers, adenosine; impaired renal function; pregnancy; cardiac arrhythmias; the presence of a pacemaker or implantable cardioverter-defibrillator; structural cardiomyopathy.

Activities of this task include execution and analysis of standard-of-care functional tests, analysis of CCTA in terms of plaque characteristics, stress CTP acquisition and interpretation. Furthermore, in patients eligible for CCTA + perfusion a blood sample will be collected before or two months after the execution of the imaging test (to avoid deleterious influences of the contrast agent) for transcriptome analysis. Whole-blood transcriptome analysis will be performed in collaboration with Centro Cardiologico Monzino. Forty patients have already been enrolled for the diagnostic pathways. We will enrol other 15 patients with stable chest pain according to the inclusion and exclusion criteria.

The following issues will be investigated:

- the prognosis of patients at 1- and 2-years comparing standard of care versus CCTA+CTP,
- correlation between high-risk plaque features, ischemia and blood transcriptional biomarkers;
- correlation between plaque characteristics and myocardial ischemia.

This study will provide information on which strategy is most effective in the diagnosis of suspected CAD in intermediate-high risk patients or symptomatic patients with known CAD and previous history of revascularization. The addition of CTP to standard CCTA provides complementary anatomical and functional information in a single examination, which may be useful for improving patient management, prediction of MACE and patient outcome. We expect to observe positive correlations between high CAD burden, ischaemia and MACE. In addition, we expect to identify a set of blood-based transcriptional biomarkers that allow accurate pre-test classification of patients with CAD into non-obstructive or obstructive CAD, negative or positive for myocardial perfusion defect.

Task 1.8. ***Integrated tools for CAD risk stratification.***

The current decision path for suspected CAD is mainly focused on the identification of high-grade coronary lumen stenosis to select patients who may benefit from myocardial revascularization. However, acute events often occur in patients who have been misclassified as "low risk" because they do not present risk factors and/or inducible myocardial ischemia. A new comprehensive approach to CAD may help to untangle its pathophysiology and may allow for early recognize individuals at higher risk. Multi-layer data, integrating clinical data (including psychological, socioeconomic, and laboratory factors), multiomics profiles, and advanced imaging analysis of coronary atherosclerosis by CCTA are needed to provide reliable predictive models of CAD, acute events, and patients' outcomes.

The main hypothesis to be tested in this part of the project is whether there are specific individual genomics/epigenomics/transcriptomics patterns whose co-occurrence with high-risk atherosclerotic plaque features as assessed at CCTA can identify different subclasses of coronary atherosclerosis or predict CAD progression and/or incident events at medium-term follow-up.

Experimental design. The full study cohort is composed of 1292 consecutive patients undergoing CCTA for clinical suspicion of CAD, without previous history of cardiovascular events, already enrolled at CCM. Inclusion criteria were (*a*) age between 18 and 80 years, (*b*) negative history of previous coronary events (angina, silent ischaemia, myocardial infarction) or revascularisation (coronary angioplasty or coronary artery bypass), and (*c*) clinical indication for CCTA to assess the presence of CAD. The exclusion criteria were pregnancy; history of previous cardiovascular events or coronary revascularisation; formal indication for immediate coronary revascularisation; arrhythmias, heart failure, non-ischemic cardiomyopathies, pacemakers, defibrillators; severe extra-cardiac pathology; immunosuppressive therapy or chemotherapy in the previous year; major surgery in the preceding six months; blood transfusions in the preceding two months. Advanced evaluation of CCTA at baseline was performed for qualitative and quantitative assessment of high-risk plaque features and functional coronary plaque assessment. Beginning this year, patients with nonobstructive or subclinical coronary atherosclerosis will undergo a second CT scan after 3-4 years from enrolment to assess coronary atherosclerosis progression. The same post-processing analysis performed for baseline CT will be repeated. During the current year, we expect to recall around 140 patients

and this will form the study cohort for this project.
Multiomics markers will be sought at baseline. They will be derived from genome-wide genotyping (which has been already performed using the Affymetrix Axiom Precision Medicine Diversity Array [PMDA] on the full cohort), genome-wide methylation analysis (which has also already been done, using the Illumina Infinium MethylationEPIC BeadChip), and whole-blood transcriptome analysis by RNA-sequencing using NGS technology. Combined with advanced coronary plaque feature detection, they will be tested for associations with the degree of plaque burden, CAD progression, and occurrence of cardiovascular events.
Non-invasive detection and early identification of high-risk phenotypes will lead to a significant change in the conventional clinical approach to CAD, moving from a lumen-centred to a comprehensive plaque and biomarker-centred approach. This will pave the way for precision medicine applications in the prevention of CAD-related events and management of CAD patients.

IRCCS involved & roles

IRCCS	Contribution of IRCCS to the realization of the WP 1 *(describe expertise applied)*	Tasks
1. Humanitas	Carotid endarterectomy. Spatial and single-cell sequencing from human atherosclerotic plaques. Gene silencing in smooth muscle cells.	1.1
2. San Donato	Carotid endarterectomy; histologic analysis; inflammatory cytokines ELISA assays; circular RNAs and inflammatory cytokine mRNA levels measurement by digital-PCR and/or RT-qPCR	1.2
3. San Martino	Carotid endarterectomy. Measurements and analysis of circulating inflammatory and calcification-mediating biomarkers.	1.3
4. San Raffaele Roma	Patient evaluation by Echo-Colour-Doppler, clinical assessment, implementation of training programs, and data management.	1.4
5. San Matteo	Coronary imaging acquisition by OCT and analysis. Definition of immuno-morpho-functional features of coronary atherosclerotic samples and thromboembolic material.	1.5
6. Gemelli	Coronary imaging acquisition by OCT and analysis. Fluid dynamics and shear stress analysis. Identification of specific biomarkers and molecular pathways in NSTEMI. Metagenomics.	1.6
7. Auxologico	Coronary imaging acquisition by CCTA and/or CT perfusion and analysis.	1.7
8. Monzino	Coronary imaging analysis by CCTA. Genome-wide genotyping, genome-wide methylation analysis, and RNA-sequencing.	1.8

Milestones
Milestone 1.1.1: Sequencing of the spatial transcriptomic processed samples & bioinformatics analysis (month 12).
Milestone 1.1.2: Gene silencing in smooth muscle cells (month 12).
Milestone 1.2.2: Vulnerable-plaque cytokine-signature identification (month 12).
Milestone 1.2.3: Vulnerable-plaque circRNA-signature identification (month 12).
Milestone 1.3.1: Quantification of FGF23/Klotho (month 8).
Milestone 1.3.2: Quantification of JCAD (month 9).
Milestone 1.3.3: Quantification of ZAG (month 10).
Milestone 1.3.4: Prognostic role of the FGF23/Klotho axis, JCAD and ZAG (month12).
Milestone 1.3.5: Enrolment of ten new patients (month12).
Milestone 1.4.1: Definition of an advanced exercise training programme in patients with carotid plaque (month 8).
Milestone 1.4.2: Assessment of changes in plaque composition after the high-intensity exercise programme (month12).
Milestone 1.5.1: Report on features of two distinct mechanisms and substrates of neoangiogenesis in human coronary atherosclerosis (month 12).
Milestone 1.6.1: Completion of OCT-based and shear stress-based analyses (month 12).
Milestone 1.6.2: Completion of biomarker-based plaque phenotyping (month 12).
Milestone 1.7.1: Stratification based on CCTA + CTP *vs.* functional tests (month 12).
Milestone 1.8.1: CCTA assessment of coronary atherosclerosis burden at baseline and follow-up (month 10).
Milestone 1.8.2: Completion of RNA-sequencing baseline assessment in the patient cohort with follow-up (month 11).
Milestone 1.8.3: Integration of multiomics and imaging biomarkers (month 12).

Expected results

Specific expected results are detailed above for each Task. Briefly, we expect to:

- ✓ Generate a comprehensive spatial transcriptomic database of human carotid atherosclerotic plaques at the single-cell level
- ✓ Identify circRNAs in PBMC and/or carotid plaques associated with vulnerability
- ✓ Identify mediators of inflammation-calcification playing a role in carotid plaque vulnerability
- ✓ Improve or halt the progression of carotid atherosclerosis through a high-intensity exercise programme
- ✓ Dissect the immuno-phenotypic and morpho-functional bases of the atherosclerotic and thrombotic/thromboembolic models of coronary plaque neoangiogenesis
- ✓ Identify patient endophenotypes of coronary instability by OCT imaging and a set of specific biomarkers in NSTEMI
- ✓ Determine which testing or imaging strategy is most effective in the diagnosis/prognosis of CAD at intermediate-high risk
- ✓ Integrate multiomics signatures and advanced CT analysis for dissecting endophenotypes of CAD burden, progression and outcomes.

Deliverables

D 1.1.1: Identification of differential cellular clusters in the analysed samples.
D 1.1.2: Identification of top-modulated genes per cell cluster.
D 1.1.3: Definition of the interaction between the different identified cellular clusters at the spatial level.
D 1.1.5: Validation of siRNA efficiency in vascular SMCs in vitro.
D 1.2.1: Dataset of inflammatory-cytokines characterizing vulnerable-plaque patients.
D 1.2.2: Dataset of atherosclerosis-circRNAs characterizing vulnerable-plaque patients.
D 1.2.3: Publication of the results by communications at National/International Meetings and in peer-reviewed journals
D 1.3.1: Database including assessment of the serum levels of FGF23/Klotho, JCAD, and ZAG.
D 1.3.2: Statistical analysis and manuscript draft.
D 1.3.3: Collection of ten novel serum and carotid plaque samples.
D 1.4.1: Individually tailored exercise training programme
D 1.4.2: Final report
D 1.5.1: Report on the prevalence of neoangiogenesis and relationship with markers of plaque haemorrhagic events in the atherosclerotic model (culprit plaques of ACS *in vivo*).
D 1.5.2: Report on the characteristics of neoangiogenesis in the thrombotic model (CTEPH).
D 1.6.1: Clinical Database Development
D 1.6.2: Biological samples collection
D 1.6.3: Diagnostic and prognostic algorithms generation, Patents
D 1.6.4: Publications in high impact journals
D 1.7.1: Report on patient stratification based on CCTA + CTP.
D 1.7.2: Report on the comparison between standard-of-care diagnostic pathway and CCTA + CTP based one.
D 1.8.1: Report on CCTA assessment of coronary atherosclerosis burden and progression.
D 1.8.2: Report on RNA-sequencing baseline data in the patient cohort with CCTA follow-up.
D 1.8.3: Report on integrative analysis.

**WP 2**	**EPIGENETIC FACTORS**
IRCCS WP leader	IRCCS Istituti Clinici Scientifici Maugeri
WP 2 Description	*Aims*<br>**Task 2.1.** To investigate noncoding RNAs (ncRNAs) and epigenetic age role in the relationship between obstructive sleep apnoea (OSA) and carotid atherosclerosis.<br>**Task 2.2.** To investigate the associations of circulating levels of P2X purinoceptor 7 (P2X7) and two microRNAs targeted by P2X7, miR-150-5p and miR-186-5p, with the clinical status of patients with carotid atherosclerosis and/or abdominal aortic aneurysm (AAA), their imaging features, and their circulating inflammatory profile.<br>**Task 2.3.** (*i*) To characterize epigenetic patterns involved in the pathogenesis and evolution of coronary atherosclerotic lesions; (*ii*) to develop an integrated multi-omics framework by combining transcriptomics

and epigenomics; (*iii*) to investigate biological interactions and networks, characterizing pathological phenotypes; (*iv*) to identify ncRNA epigenetic modifications in the pathophysiology of atherosclerosis.
Task 2.4. Identification of microRNAs associated with coronary atherosclerosis, both circulating in coronary and venous blood and expressed in cells obtained from stable and unstable atherosclerotic plaques, to contribute to the estimation of acute coronary event risk.
Task 2.5. To identify molecular signatures of circulating exosomal microRNAs in patients at risk for atherosclerotic cardiovascular disease (coronary disease).

start date-end date: **01/01/2022 – 31/12/2022**

Activities of the WP

Task 2.1. ***Role of epigenetic factors in the interplay between obstructive sleep apnoea and atherosclerosis.***
In a previous study, we tested the hypothesis that OSA-related candidate miRNAs (miR-485-5p, miR-139-3p, miR-574-5p, and miR-107) would be differentially expressed in patients with/without OSA and carotid atherosclerosis. Treatment with continuous positive airway pressure (CPAP) restored circulating miR-485-5p expression. miR-139-3p was not affected by OSA but only by carotid plaque.
The modulation of miR-485-5p, which seems to be exceptionally sensitive to oxygen variation, will be compared to an already known oxygen-sensible miRNA (miR-210-3p), which will be used as a positive control. Since bioinformatics on miR-485-5p revealed a solid and interactive correlation with the long noncoding RNAs (lncRNAs) MALAT1 and BACE1-AS, we will address (*a*) the impact of the MALAT1/miR-485-5p and BACE1-AS/miR-485-5p axes in OSA with/without carotid atherosclerotic plaque and (*b*) their modulation by biological ageing (in terms of DNA methylation age, DNAmAge, and DeltaAge). The evaluation of DNAmAge and DeltaAge in patients with severe OSA before and after CPAP might be useful to better understand the ageing process and their influence on carotid atherosclerosis.

Task 2.2. ***P2X7 as a risk marker for the development of abdominal aortic aneurysm in patients with carotid atherosclerosis.***
1. Enrolment and characterization (clinical features, ECG, and imaging by supra-aortic trunk Echo-Colour-Doppler and thoracic X-ray) of a cohort of patients with AAA undergoing aneurysmectomy, without carotid plaques (CPL) (group 0, n=10), or with CPL with a stenosis degree <40% (group 1, n=10, 7 of them already enrolled), or with CPL with a stenosis degree > 40% (group 2, n=10, 5 of them already enrolled), and a cohort with CPL but without AAA (group 3, n=10 to be enrolled). Patients previously undergoing endarterectomy will be excluded. Enrolment of matched controls, i.e. volunteers that will auto-certify their healthy status (n=10, already enrolled).
2. Creation of an anonymised database collecting patient' data.
3. Sampling of blood and AAA tissue from patients undergoing surgery.
4. Processing of blood samples to obtain serum.
5. Profiling of inflammation-related cyto/chemokines (>15) in the patient serum.
6. Determination of circulating P2X7 protein amount by specific ELISA and of *P2X7*, miR-150-5p, and miR-186-5p expression by RT-qPCR,
7. RNA isolation from AAA tissues and determination of *P2X7*, miR-150-5p, and miR-186-5p expression by RT-qPCR and correlation with circulating and tissue levels.
8. Statistical analysis of the relationships between clinical and experimental data.

This activity will obtain proof of concept data on whether and how P2X7 and two target miRs differ between patients with both AAA requiring surgery and CPL and those with only one of these vascular lesions, also in comparison with normal controls. Patients with CPL are shown to be at higher risk of incident clinical AAA (see the ARIC study) and, although atherosclerosis is not the cause of AAA, we expect that CPL presence affects the inflammatory profile of patients with AAA and increases the amount of shed P2X7. Because P2X7 may indirectly regulate the proliferation of SMCs, important for the AAA growth, through two target miRs, miR-150-5p and miR-186-5p, we expect to find more elevated levels of these microRNAs either in the serum or the tissue of patients demonstrating a worsening clinical status and/or biggest AAA. If the results confirm this hypothesis, our data will indicate P2X7 as a putative biomarker for AAA risk, to be further investigated in larger cohorts.

Task 2.3. ***Epigenetic hallmarks discovery in coronary atherosclerosis.***
The epigenome provides a mechanistic link between environmental exposures and gene expression profiles ultimately leading to disease. Characterization of these epigenetic differences may enable the identification of key genes and biological mechanisms involved in atherosclerotic cardiovascular disease. The primary objective is the identification, through NGS integrated analysis supported by RT-qPCR validation, of potential regulatory elements active in the genome starting from regions of accessible chromatin, which can contribute to highlighting peculiar epigenetic alterations in the vessel walls subjected to atherosclerotic stress, to identify potential new biomarkers of diagnostic and/or prognostic interest. A further objective is to evaluate the possible association or correlation of epigenetic alterations with demographic and clinical characteristics as well as with features of available imaging data.
Specifically, this task will develop a multiomics approach for the evaluation and the characterization, in a clinical setting, of the epigenetic profile from mononuclear cells in peripheral blood samples (PBMC) obtained from patients with a diagnosis of CAD ascertained based on diagnostic examinations (imaging tests) performed in house, such as CCTA, myocardial perfusion scintigraphy, PET-TAC and/or PET MRI. Activities will include: data processing and feature extraction from diagnostic images; library preparation for ATAC-sequencing and RNA-sequencing experiments and validation of putative epigenetic biomarkers; bioinformatics techniques for NGS data analysis and multiomics data integration, and network-based approaches to support precision medicine.

Task 2.4. ***Circulating and cellular microRNAs in coronary atherosclerosis.***
The role of several miRNAs in the regulation of different biological pathways involved in atherosclerosis development and progression has been described using preclinical models. Altered expression of different circulating miRNAs in the presence of stable atherosclerosis of specific territories has been identified in humans. We previously identified circulating microRNAs involved in the modulation of inflammatory status, the inflammamiRs (Olivieri 2021), and related to cardiomyocyte necrosis (Olivieri 2014). However, to the best of our knowledge, there are no published data on the relationship between the expression of inflammation-related microRNAs, i.e. miR-146a and miR-21, and cardiomyocyte-associated microRNAs, i.e. miR-499, in coronary and venous blood from patients affected by acute or chronic coronary syndromes. This task will set the basis for a three-year project, by establishing a protocol with other IRCCS of the network for the study of microRNAs associated with coronary atherosclerosis, circulating in coronary and venous blood and expressed in cells obtained from stable and unstable atherosclerotic plaques. The protocol will also include the measurement of Advanced Glycation end products (AGE) and the soluble forms of AGE receptor (sRAGE) in the blood of enrolled patients. The ability of selected microRNAs and inflammatory markers to contribute to the estimation of the risk of acute coronary events, in association with imaging and clinical data, will be analysed. The protocol will be sent for approval to the Ethical Committees and IRCCS INRCA will then perform a small pilot study to validate the protocol given performing a larger study in the following three years.
2.4.1. Protocol and Ethical approval. The protocol will describe the type of patients (stable and unstable), the methodology for collecting the plaque samples, and how samples will be handled, treated, and analysed.
2.4.2. Pilot study. A small pilot study will be performed on 10 patients to assess the validity of the protocol. The protocol will then be modified accordingly.

Task 2.5. ***Identification of exosomal microRNA with a prognostic value for coronary atherosclerosis.***
This task aims to identify molecular signatures of circulating biomarkers, in particular exosomal microRNAs, associated with atherosclerotic cardiovascular disease (ASCVD) with a particular focus on coronary atherosclerosis and related conditions. The samples of patients with coronary atherosclerosis will be provided by the IRCCS Centro Cardiologico Monzino and IRCCS ISMETT will perform the molecular measurement of circulating microRNAs. The results obtained will be analysed by differential analysis, unsupervised hierarchical clustering, and principal component analyses (PCA). The ultimate goal remains to identify epigenetic biomarkers capable of "predicting" the presence of plaque and therefore the presence of risk for spontaneous cardiovascular events. We will also evaluate correlations between the composition of the circulating miRNoma and quantitative/qualitative indices of coronary stenosis.
Specific activities will be:

	- Patient enrolment; storage and shipment of the samples.<br>- microRNA signatures assessments.<br>- Analysis of the results for the correlation with the extent and features of coronary atherosclerotic disease.

IRCCS involved & roles

IRCCS	Contribution of IRCCS to the realization of the WP 2 *(describe expertise applied)*	Tasks
1. Maugeri	Assessment of epigenetics, lncRNA, miRNA, DNAmAge, and daytime sleepiness.	2.1
2. San Raffaele MI	Sampling and processing of blood and AAA tissue; microRNA expression analysis.	2.2
3. SYNLAB SDN	Diagnostic imaging; NGS and qPCR validation; multiomics data integration.	2.3
4. INRCA	Protocol definition and coordination of activities; miRNA detection.	2.4
5. Monzino	Patient enrolment; CCTA image acquisition; patient stratification according to CCTA features.	2.5
6. ISMETT	Identification of risk exosomal microRNA signatures of coronary atherosclerotic disease.	2.5

Milestones

Milestone 2.1.1: Identification of CPAP therapy effects on the expression of candidate OSA-related miRNAs (m 12).
Milestone 2.1.2: Differential expression of lncRNA in severe OSA and/or carotid plaques (month 12).
Milestone 2.1.3: Influence of CPAP treatment on epigenetic age in patients with severe OSA and/or carotid plaques (m12).
Milestone 2.2.1: Completion of recruitment and characterization of patients and controls (month 8).
Milestone 2.2.2: Completion of sample processing and experimental analyses (month 10).
Milestone 2.2.3: Complete statistical analyses on associations between patients' status and measured variables (month 12).
Milestone 2.3.1: Inclusion and exclusion criteria from imaging for NGS experiments (month 6).
Milestone 2.3.2: Sequencing experiments of ATAC-seq and RNA-seq (month 8).
Milestone 2.3.3: NGS data analysis and multi-omics data integration (month 10)
Milestone 2.3.4: Experimental validations and clinical implication (month 12).
Milestone 2.4.1: Protocol approved by Ethical Committee (month 10).
Milestone 2.4.2: Pilot study completed (month 12).
Milestone 2.5.1: Project partner meeting for the definition of experimental timeline and analysis (month 1).
Milestone 2.5.2: Final results report (month 12).

Expected results

Specific expected results are detailed above for each Task. Briefly, we expect to:

- ✓ Clarify the role of specific microRNAs, lncRNAs, and DNA methylation age in OSA/atherosclerosis interaction
- ✓ Obtain proof of concept that P2X7 and two target microRNAs are biomarkers of the risk of AAA in patients with carotid atherosclerosis
- ✓ Characterization of the variations in chromatin accessibility in PBMC of CAD patients
- ✓ Obtain proof of concept that cellular or circulating inflammamiRs are associated with coronary atherosclerosis type
- ✓ Identify exosomal microRNAs with prognostic values for different endophenotypes of CAD.

Deliverables

D 2.1.1: Validation of the effects of CPAP treatment on miR-485-5p expression level compared to the expression level of miR210-3p.
D 2.1.2: Demonstration of MALAT1 and BACE1-AS upregulation in OSA patients with/without carotid plaques and their modulation after CPAP.
D 2.1.3: Identification of biological age (DNAmAge) in patients and controls.
D 2.1.1: Patient database.
D 2.2.2: Dataset on inflammatory status, P2X7, and target microRNA expression in patients' blood and AAA tissue.
D 2.2.3: Results on the association between P2X7 and target miRs, inflammatory profile and clinical status of the patients.
D 2.3.1: Results on epigenetic alterations as potential targets for planning future strategies to improve patient diagnosis and/or therapy.
D 2.3.2: Insight into the inflammatory mechanisms contributing to the formation of atherosclerotic plaques and involved in the cardiovascular physiopathology.
D 2.4.1: Definition of a protocol for the identification of microRNAs associated with coronary atherosclerosis, both circulating in coronary and venous blood and expressed in cells obtained from stable and unstable atherosclerotic plaques.

D 2.5.1: Identification of circulating biomarkers associated with the presence of atherosclerotic cardiovascular disease.
D 2.5.2: Identification of risk signatures for coronary disease.
D 2.5.3: Interpretation and prediction of the associated gene pathways for disease onset.

**WP 3**	**IMMUNOLOGICAL COMPONENTS**
IRCCS WP leader	IRCCS MultiMedica
WP 3 description	*Aims*<br>**Task 3.1.** (*i*) Profiling Natural Killer (NK) cell subset distribution (circulating and plaque infiltrating) in peripheral artery atherosclerosis and carotid atherosclerosis. (*ii*) Investigating the functional crosstalk between NK cells and relevant atherosclerosis plaque cellular components (monocytes/macrophages and endothelial cells).<br>**Task 3.2.** Characterization of the subpopulations, localization, and autophagic profile of immune cells infiltrating the atherosclerotic plaque of patients with critical carotid artery stenosis.<br>**Task 3.3.** Study of the molecular forms of intraplaque ficolin-2 to help the identification of pharmacological approaches to inhibit it. Clarification of the main molecular cascades activated downstream to ficolin-2 in the atherosclerotic carotid plaque.
	start date-end date: **01/01/2022 – 31/12/2022**
	Activities of the WP<br><br>**Task 3.1.** ***NK cells and carotid and/or femoral atherosclerosis.***<br>Exacerbated inflammation and immune cell polarization can be considered relevant host-dependent hallmarks of the atherosclerotic plaques. Monocytes/macrophages and T cells have been the classical prototype of immune cells investigated in the atherosclerotic context. NK cells, i.e., innate lymphoid cells (ILCs) primarily involved in tumour cell recognition and elimination, have recently been proposed to play an immunoregulatory role in atherosclerosis (ATS) pathogenesis/progression. Although several studies have evaluated the frequency and/or function of NK cells in human and animal models of ATS, due to conflicting results it is still unclear whether circulating and ATS plaque-infiltrating NK cells act as protective or proatherogenic effectors. We hypothesized that: (*i*) phenotype and functional alterations of NK cells in the plaque tissues and peripheral blood of symptomatic ATS patients significantly impact ATS fate/progression; (*ii*) NK cell alteration in ATS patients can instruct other cellular effectors (i.e., monocyte/macrophages, endothelial cells) to be supportive in ATS progression/fate.<br><u>*3.1.1 Experimental design Aim (i).*</u> Clinical samples will include heparinized-collected peripheral blood (20 ml/donor) and plaque tissue from patients hospitalized for ATS disease and undergoing carotid (EAC) or femoral (TEA femoral) endarterectomy procedures. Peripheral blood samples from healthy age-matched donors will be used as controls. NK cell phenotyping/subset distribution will be performed by multicolour flow cytometry, on total mononuclear cells, isolated from peripheral blood (subjected to Ficoll stratification) and enzymatically processed ATS plaques. Antigens detected will include CD56, CD16, NKG2D, CD69 (marker of activation), CD57 (maturation), CD9, CD49a (decidual-like markers). Th1, Th2 and Th17-like cytokine production/release will be assessed on conditioned media from FACS-sorted NK cells, using commercially available secretome membrane arrays and Bioplex. Results from NK immunophenotyping and the related cytokines milieu will be correlated with clinical parameters (duration of disease, severity of symptoms, anthropometric data, comorbidities, cardiovascular risk factors such as lifestyle, smoking, alcohol consumption and dietary habits, information on chronic medications, blood pressure, waist circumference, BMI), to explore whether ATS patients can be stratified by the NK cell polarization state.<br><u>*3.1.2. Experimental design Aim (ii).*</u> CD14+ derived-M0 macrophages or endothelial cells will be co-cultured with FACS-sorted circulating or plaque-infiltrating NK cells from ATS patients or their

conditioned media, to investigate whether direct and/or paracrine interactions would impact their crosstalk. PINK/PANK-derived conditioned media will be used to perform functional angiogenesis assays to investigate endothelial (microvascular and human umbilical vein) proliferation, chemotaxis, morphogenesis and sprouting. Monocytes will be isolated from the peripheral blood of control subjects, by magnetically positive selection of total CD14+ cells. CD14+ derived macrophages will be generated by differentiating CD14+ monocytes with M-CSF (50 ng/mL), for 1 week. The effects of circulating or plaque infiltrating NK cells from ATS patients, either as a co-culture system or using their conditioned media, will be tested by qPCR, to assess macrophage polarization (M1 Vs M2 markers).

Power calculation and statistical analysis. We estimated that a sample size of 50 ATS patients and 50 age-matched healthy controls will achieve 85% power to establish a 30% rate of difference between the two groups analysed (two-sided t-test with a significance level alpha of 5%). Statistical differences between two datasets will be determined using a two-tailed Student’s t-test for continuous variables and the chi-squared test (or Fisher's exact test) for categorical variables. Analysis of multiple data sets will be performed by one-way ANOVA and/or two-way ANOVA. P-values <0.05 will be considered statistically significant.

The main expected results from this task will be (1) the identification of specific surface antigens/cytokine profiling able to dynamically trace major alteration of NK cells, contributing to ATS progression; (2) the definition of shared or different NK cell subsets distribution/function in peripheral blood and the plaque (local tissue). Also, results obtained would provide significant advancement over the current state of the art and clinical translation/application on NK cells in the context of NK cell biology in ATS, by allowing (i) the identification of a cellular biomarker (altered NK cell phenotype), detectable with a minimally invasive procedure (liquid-based biopsy); (ii) identifying potentially NK cell-oriented-targets for possible (immune) therapy in ATS; (iii) deliver novel experimental tools, to be translated to the clinic and providing new insights regarding pro-atherogenic activities by circulating or plaque infiltrating NK cells in ATS patients.

Task 3.2. *Neuroimmune factors in carotid plaque vulnerability.*

Recent evidence in experimental models of atherosclerosis indicates that the progression of the disease and the atherosclerotic plaque stability depend on neuroimmune factors linked not only to the local microenvironment of the plaque but also to a fine balance of homeostatic systems such as the autonomic nervous system (Mohanta 2022). As the disease progresses, adventitial immune cells infiltrate and expand systemically in the arterial tree and some immune cell aggregates develop into well-structured artery tertiary lymphoid organs (ATLOs) at distinct sites in both mouse and human arterial beds (Mohanta 2014; Akhavanpoor 2014; Brunet 2014). More interesting, the autonomic nervous system interacts with plaque-associated adventitial leukocytes and the denervation of the sympathetic nervous system (SNS) disrupts ATLOs and attenuates atherosclerosis progression (Mohanta 2022). Furthermore, recent evidence suggests that a dysregulation of the autophagic process determines the activation of the inflammatory immune response during the atherosclerosis process (Sergin 2017).

To identify correlations between immune response, the autonomic nervous system activation, and the autophagic process activated during the progression of atherosclerosis, in this project, we will analyze histological samples already collected in our previous studies to:

1) Characterize and localize the immune cells in atherosclerotic plaque by immunohistochemistry. In particular, we will characterize the immune cells infiltrating and aggregating in atherosclerotic plaques and we will identify the localization of immune cells around the plaque to investigate which immune cells participated in arterial wall remodelling during the disease and where adaptive immune responses are organized. We will also deepen the contributions of innate immunity carried out by subtypes of blood-derived monocyte/macrophages and foam cells, dendritic cells (DC) subtypes, and B cells.
2) Analyse the autophagic profile of immune cells activated during the atherosclerotic process;
3) Verify the presence of sympathetic/parasympathetic autonomic nervous system innervation in correspondence with specific immune cell subtypes. In particular, we will quantify and analyse the NF200+ and TH+ axons in the plaques by immunofluorescence analyses to investigate whether an increase of sympathetic innervation in atherosclerotic plaque could be correlated with an increased presence of infiltrating adaptive immune cells.

Atherosclerosis is now categorized as a chronic inflammatory disease of the arterial wall with a significant immune component. These activities will allow us to analyse immune cells infiltrating and aggregating in

atherosclerotic plaques to evaluate the localization of specific subtypes in arterial wall remodelling. Moreover, tissue biopsy analyses will be fundamental to evaluate the involvement of the autonomic nervous system in the crosstalk with specific immune cell subpopulation in atherosclerotic plaques, to correlate the neuroimmune activation with plaque stability/instability and clinical impairment. The expression levels of the autophagic markers will be correlated with markers of neuroimmune activation and with the characteristics of the plaque. Delineation of immune cell subtypes and innervation in atherosclerotic plaques may provide clues for further research into human atherosclerosis opening unprecedented avenues for immune-based therapeutics.

Task 3.3. ***Intraplaque molecular mechanisms associated with the initiator of the lectin pathway of the complement system ficolin-2.***

This task is a continuation of the activities undertaken during the previous projects, having successfully identified in ficolin-2 a circulating biomarker of the vulnerable carotid plaque (PMID: 33513354). High levels of circulating ficolin-2 - measured 1 day before surgery in atherosclerotic patients eligible for endarterectomy - are associated with plaques with a vulnerable morphology, characterized by ulceration and alteration of the intima-media thickness. Plaques with this morphology bear an increased risk of thromboembolic complications, including stroke. Ficolin-2 was found associated with intraplaque cholesterol crystals and infiltrated immune cells. Besides its use as a biomarker, ficolin-2 may be an attractive pharmacological target to dampen plaque vulnerability in atherosclerotic patients. Putative drugs already in clinical trials are monoclonal antibodies directed toward the serine proteases associated with ficolin-2, the so-called MASPs, that are needed to activate the full complement cascade. However, our previous data do not clarify if intraplaque ficolin-2 is linked to MASPs, thus locally activating the full complement cascade, or is rather present in mono or multimeric forms directly targeting cholesterol crystals and immune cells. Recognition molecules of the complement system, like ficolin-2, acquire different oligomerization states to allow selective target binding through an avidity-based mechanism. As such knowing the intraplaque ficolin-2 oligomerization state may inform about its function. The main aim of this project task will be that of describing the molecular forms of intraplaque ficolin-2 to help the identification of pharmacological approaches to inhibit it. We will then clarify the main molecular cascades activated downstream to ficolin-2 in the atherosclerotic carotid plaque.

Specific activities include:

3.3.1. Homogenates of plaque specimens will be processed for non-reducing Tris-acetate buffer Western blot, able to distinguish multimeric forms of ficolin-2 and their selective binding to MASPs. (MASP-1, -2 and -3).

3.3.2. Immunoprecipitation assays will be used to identify and measure ficolin-2-MASPs molecular binding in plaque homogenates.

3.3.3. Immunofluorescence and confocal microscopy will be used to study ficolin-2-MASPs co-localization in the histological specimens.

3.3.4. Relevant protein-protein interactions identified in the previous tasks will be studied in blood from the same patients, analysing their longitudinal changes, i.e., 1 day before and 2 days after endarterectomy.

3.3.5. New patients will be enrolled to obtain fresh tissues and clinical information (including if experiencing COVID-19 positivity over the previous 6 months before surgery, as the infection may alter MASPs), to obtain a larger group size fostering more solid data.

We expect to define whether ficolin-2 intraplaque mechanisms depend on its direct chemotactic functions towards immune cells and/or on the activation of the lectin pathway of complement through the MASPs serine proteases. Once defined the molecular forms of intraplaque ficolin-2, we will be able to provide information on the most promising pharmacological approach to target intraplaque ficolin-2 functions.

IRCCS involved & roles

IRCCS	Contribution of IRCCS to the realization of the WP 3 *(describe expertise applied)*	Tasks
1. MultiMedica	Collection of blood samples and plaque specimens. Phenotype and functional characterization of circulating and ATS plaque infiltrating NK cells by multicolour flow cytometry, cell sorting and studies of cell-to-cell and soluble factors-cell interactions (with monocytes/macrophages and endothelial cells).	3.1
2. Neuromed	Immunophenotyping and localization of immune cells infiltrating and aggregating in atherosclerotic plaques during arterial wall remodelling. Analysis of markers of autophagy in tissue biopsy. Examining the presence and quantification of NF200+ and TH+ axons in the plaques.	3.2
3. Mario Negri	Analyses of the forms of Ficolin-2, binding to MASPs, and protein-protein interactions. Histology.	3.3.1-3.3.4
4. Mondino	New patient enrolment and tissue and clinical data collection.	3.3.5

Milestones
Milestone 3.1.1: Identification of surface antigens and cytokine repertoire in pro-atherogenic circulating and plaque-infiltrating NK cells (month 10).
Milestone 3.1.2: Identifications of circulating NK cells from ATS patients as peripheral/systemic surrogates of plaque infiltrating NK cells (month 10).
Milestones 3.1.3 Identification of NK cell regulatory functions within their crosstalk with endothelial cells and monocytes/macrophages in the ATS context (month 12).
Milestone 3.2.1: Identify and localize the immune cells infiltrating atherosclerotic plaques by immunofluorescence and immunohistochemistry analyses (month 4).
Milestone 3.2.2: Analysis of the expression levels of the autophagic markers in tissue biopsy (month 7).
Milestone 3.2.3: Analysis of the neuroimmune activation by quantification of NF200+ and TH+ axons in the plaques (month 12).
Milestone 3.3.1: Definition of the molecular forms and interactions of intraplaque ficolin-2 (month 10).
Milestone 3.3.2: Definition of time-dependent changes of the molecular forms and interactions of circulating ficolin-2 (month 12).

Expected results
Specific expected results are detailed above for each Task. Briefly, we expect to:
- ✓ Clarify the role of circulating or infiltrating NK cell subsets in carotid atherosclerosis progression
- ✓ Characterize neuroimmune components (infiltrating immune cells, autophagic profile, sympathetic/parasympathetic innervation and their interaction) in carotid plaque vulnerability
- ✓ Define whether ficolin-2 actions in carotid plaques depend upon its chemotactic ability or the activation of the lectin pathway of the complement system.

Deliverables
D 3.1.1: Surface antigens panel characterizing circulating or plaque infiltrating NK cells from ATS patients.
D 3.1.2: Specific cytokine signature/panel characterizing circulating or plaque infiltrating NK cells from ATS patients.
D 3.1.3: Circulating NK cells from ATS patients as peripheral/systemic surrogates of plaque infiltrating NK cells.
D 3.1.4: Cellular interactors in the crosstalk between circulating or plaque infiltrating NK cells, from ATS patients.
D 3.2.1: Contribution of immune cell subpopulations in the arterial wall remodelling process.
D 3.2.2: Correlation of the autophagic process with inflammatory immune response activation.
D 3.2.3: Correlation of the neuroimmune activation with plaque stability/instability and clinical impairment.
D 3.3.1: Report on ficolin-2-MASPs complexes in the atherosclerotic plaque.
D 3.3.2: Report on ficolin-2 oligomerization states in the atherosclerotic plaque
D 3.3.3: Time-dependent changes in circulating ficolin-2 molecular forms in the atherosclerotic patients.

Section C	BUDGET PROPOSAL

TOTAL PROPOSED BUDGET			
**COST** *(categories)*	TOTAL BUDGET **(€)** **A=B+C**	PROJECT COSTS PROPOSED FOR FUNDING TO THE MOH **(€)** **B** *The amounts must be reported without the decimal fraction.*	CO-FUNDING *if applicable* **(€)** **C**
staff salary	294.400,00	294.400,00	0
research contracts	287.000,00	287.000,00	0
travel	20.550,00	20.550,00	0
equipment (leasing-rent)	50.000,00	50.000,00	0
supplies and materials	787.150,00	787.150,00	0
publication costs	33.300,00	33.300,00	0
conferences	8.750,00	8.750,00	0
staff training	8.500,00	8.500,00	0
subcontracts	39.000,00	39.000,00	0
other costs	31.000,00	31.000,00	0
overhead	168.370,00	168.370,00	0
**total**	**1.728.020,00**	**1.728.020,00**	0

COSTS STRUCTURE PER EACH UNIT-IRCCS

N.	Name	Fiscal code number	Public-private	(€) *The amounts must be reported without the decimal fraction.*
1	IRCCS Centro Cardiologico Monzino	13055640158	private	224.000,00
2	IRCCS Fondazione Policlinico San Matteo	00303490189	public	71.270,00
3	IRCCS Istituti Clinici Scientifici Maugeri S.p.A. Società Benefit	02631650187	private	95.000,00
4	IRCCS Istituto Clinico Humanitas	10125410158	private	95.000,00
5	IRCCS Istituto di Ricerche Farmacologiche Mario Negri	03254210150	private	95.000,00
6	IRCCS San Raffaele Roma	10636891003	private	95.000,00
7	ISMETT Istituto Mediterraneo per i Trapianti e Terapie ad Alta Specializzazione	04544550827	private	95.000,00
8	IRCCS Istituto Neurologico Mediterraneo, NEUROMED	00068310945	private	125.000,00
9	IRCCS Fondazione Istituto Neurologico Nazionale C. Mondino	00396070187	private	95.000,00
10	IRCCS Ospedale Policlinico San Martino	02060250996	public	95.000,00
11	IRCCS Ospedale San Raffaele	07636600962	private	95.000,00
12	IRCCS MultiMedica	06781690968	private	95.000,00

13	Fondazione Policlinico Universitario Agostino Gemelli IRCCS	13109681000	private	95.000,00
14	IRCCS Istituto Auxologico Italiano	02703120150	private	95.000,00
15	IRCCS Policlinico San Donato	05853360153	private	97.750,00
16	IRCCS SDN Synlab	01288650631	private	70.000,00
17	IRCCS Istituto Nazionale di Riposo e Cura per Anziani - INRCA	00204480420	public	95.000,00

**Section D**	**REFERENCES**
1	*Joehanes, R. et al. Gene expression analysis of whole blood, peripheral blood mononuclear cells, and lymphoblastoid cell lines from the Framingham Heart Study. Physiological genomics 44, 59-75, doi:10.1152/physiolgenomics.00130.2011 (2012).*
2	*Khyzha, N et al. Epigenetics of Atherosclerosis: Emerging Mechanisms and Methods. Trends Mol. Med. 23, 332-347, doi: 10.1016/j.molmed.2017.02.004 (2017).*
3	*Ridker, P. M. et al. Antiinflammatory Therapy with Canakinumab for Atherosclerotic Disease. N Engl J Med 377, 1119-1131, doi:10.1056/NEJMoa1707914 (2017).*
4	*Farina F, Hall F, Serio S, Zani S, Climent M, Civilini E, Condorelli G, Quintavalle M and Elia L. "The epigenetic enzyme DOT1L orchestrates vascular smooth muscle cell monocyte crosstalk and protects against atherosclerosis via the NF-kappa B pathway". European Heart Journal, 2022. Doi: 10.1093/eurheartj/ehac097.*
5	*Hall IF, Climent M, Viviani CA, Papa L, Tragante V, Farina FM, Kleber ME, März W, Biguori C, Condorelli G and Elia L. "rs41291957 variant controls miR-143 and miR-145 expression and impacts Coronary Artery Disease risk". EMBO Molecular Medicine, 2021. Doi: 10.15252/emmm.202114060.*
6	*Geovanini, G.R.; Libby, P. Atherosclerosis and Inflammation: Overview and Updates. Clin. Sci. (Lond) 2018, 132, 1243-1252.*
7	*Shimada, K. Immune System and Atherosclerotic Disease: Heterogeneity of Leukocyte Subsets Participating in the Pathogenesis of Atherosclerosis. Circ. J. 2009, 73, 994-1001*
8	*Puig, N.; Jimenez-Xarrie, E.; Camps-Renom, P.; Benitez, S. Search for Reliable Circulating Biomarkers to Predict Carotid Plaque Vulnerability. Int. J. Mol. Sci. 2020, 21, 10.3390/ijms21218236.*
9	*Carrara M, Fuschi P, Ivan C, Martelli F. Circular RNAs: Methodological challenges and perspectives in cardiovascular diseases. J Cell Mol Med. 2018 Nov;22(11):5176-5187. doi: 10.1111/jcmm.13789.*
10	*Gomes, C.P.C.; Schroen, B.; Kuster, G.M.; Robinson, E.L.; Ford, K.; Squire, I.B.; Heymans, S.; Martelli, F.; Emanueli, C.; Devaux, Y. et al. Regulatory RNAs in Heart Failure. Circulation 2020, 141, 313-328.*
11	*Holdt, L.M.; Kohlmaier, A.; Teupser, D. Molecular Functions and Specific Roles of circRNAs in the Cardiovascular System. Noncoding RNA Res. 2018, 3, 75-98*
12	*Carbone et al. Serum levels of osteopontin predict major adverse cardiovascular events in patients with severe carotid artery stenosis. Int J Cardiol. 2018;255:195-9*
13	*Bonaventura et al. Platelet-to-lymphocyte ratio at the time of carotid endarterectomy is associated with acute coronary syndrome occurrence. J Cardiovasc Med. 2019*
14	*Montecucco F, et al. Systemic and intraplaque mediators of inflammation are increased in patients symptomatic for ischemic stroke. Stroke. 2010;41:1394-404.*
15	*Montecucco F, et al. Anti-Apolipoprotein A-1 auto-antibodies are active mediators of atherosclerotic plaque vulnerability. Eur Heart J. 2011;32(4):412-21.*
16	*Dimitrijevic O, et al. Serial measurements of C-reactive protein after acute myocardial infarction in predicting one-year outcome. Int Heart J. 2006;47:833-42.*
17	*Gordin D, et al. Osteopontin is a strong predictor of incipient diabetic nephropathy, cardiovascular disease, and all-cause mortality in patients with type 1 diabetes. Diabetes Care. 2014;37:2593-600.*
18	*Kanbay M, et al. Serum sclerostin and adverse outcomes in non-dialyzed chronic kidney disease patients. J Clin Endocrinol Metab. 2014;99:E1854-61.*

19	*Arbustini E, Morbini P, D'Armini AM, Repetto A, Minzioni G, Piovella F, Viganó M, Tavazzi L. Plaque composition in plexogenic and thromboembolic pulmonary hypertension: the critical role of thrombotic material in pultaceous core formation. Heart. 2002 Aug;88(2):177-82. doi: 10.1136/heart.88.2.177. PMID: 12117850; PMCID: PMC1767204.*
20	*Kodama T, Narula N, Agozzino M, Arbustini E. Pathology of plaque haemorrhage and neovascularization of coronary artery. J Cardiovasc Med (Hagerstown). 2012 Oct;13(10):620-7. doi: 10.2459/JCM.0b013e328356a5f2. PMID: 22828775.*
21	*Michel JB, Virmani R, Arbustini E, Pasterkamp G. Intraplaque haemorrhages as the trigger of plaque vulnerability. Eur Heart J. 2011 Aug;32(16):1977-85, 1985a, 1985b, 1985c. doi: 10.1093/eurheartj/ehr054. Epub 2011 Mar 12. PMID: 21398643; PMCID: PMC3155759.*
22	*Arbustini E, Urtis M, Prati F. OCT/atherectomy/pathology studies open new perspectives for in vivo characterization of plaque composition. Int J Cardiol. 2019 Jun 1;284:14-15. doi: 10.1016/j.ijcard.2018.10.105. Epub 2018 Oct 30. PMID: 30414747.*
23	*Arbustini E, Kodama T, Prati F. Similar Plaque Composition in Men and Women With Stable CAD: Another Myth Falls. JACC Cardiovasc Imaging. 2016 Apr;9(4):408-10. doi: 10.1016/j.jcmg.2016.02.014. PMID: 27052269.*
24	*Arbustini E, Morbini P, D'Armini AM, Repetto A, Minzioni G, Piovella F, Viganó M, Tavazzi L. Plaque composition in plexogenic and thromboembolic pulmonary hypertension: the critical role of thrombotic material in pultaceous core formation. Heart. 2002 Aug;88(2):177-82. doi: 10.1136/heart.88.2.177. PMID: 12117850; PMCID: PMC1767204.*
25	*Araki M, Park SJ, Dauerman HL, et al. Optical coherence tomography in coronary atherosclerosis assessment and intervention [published online ahead of print, 2022 Apr 21]. Nat Rev Cardiol. 2022;10.1038/s41569-022-00687-9. doi:10.1038/s41569-022-00687-9*
26	*Olivieri F, Prattichizzo F, Giuliani A, Matacchione G, Rippo MR, Sabbatinelli J, Bonafè M. miR-21 and miR-146a: The microRNAs of inflammaging and age-related diseases. Ageing Res Rev. 2021 Sep;70:101374. doi: 10.1016/j.arr.2021.101374*
27	*Olivieri F, Antonicelli R, Spazzafumo L, Santini G, Rippo MR, Galeazzi R, Giovagnetti S, D'Alessandra Y, Marcheselli F, Capogrossi MC, Procopio AD. Admission levels of circulating miR-499-5p and risk of death in elderly patients after acute non-ST elevation myocardial infarction. Int J Cardiol. 2014 Mar 15;172(2):e276-8. doi: 10.1016/j.ijcard.2013.12.203*
28	*Akhavanpoor, M. et al. Adventitial inflammation and its interaction with intimal atherosclerotic lesions. Front. Physiol. 5, 296 (2014).*
29	*Brunet, I. et al. Netrin-1 controls sympathetic arterial innervation. J. Clin. Invest. 124, 3230–3240 (2014).*
30	*Mohanta, SK, et al. Artery tertiary lymphoid organs contribute to innate and adaptive immune responses in advanced mouse atherosclerosis. Circ. Res. 114, 1772–1787 (2014).*
31	*Mohanta SK, et al. Neuroimmune cardiovascular interfaces control atherosclerosis. Nature. May;605(7908):152-159 (2022).*
32	*Sergin I, et al. Exploiting macrophage autophagy-lysosomal biogenesis as a therapy for atherosclerosis. Nat Commun. 2017 Jun 7;8:15750 (2017).*
33	*Carbone et al., Pharmacol Res. 2021 Apr;166:105462. doi: 10.1016/j.phrs.2021.105462. Epub 2021 Jan 26. PMID: 33513354*

NETWORK<br>Rete Cardiologica	Legal Representative [1]	*Dr. Lorenzo Menicanti*
	Scientific coordinator[2]	*Dr. Lorenzo Menicanti*
IRCCS Project Leader<br>Centro Cardiologico<br>Monzino IRCCS	Legal Representative[3]	*Ing. Mauro Melis*
	Scientific coordinator[4]	*Prof. Giulio Pompilio*

[1] Digital signature
[2] Digital signature
[3] Digital signature
[4] Digital signature

Ministero della Salute

WORKFLOW DELLA RICERCA 2.0

Riepilogo comunicazione

**Risposta al messaggio**	
**ID invio**	2022011816
**Data**	30/09/2022 14:51
**Stato**	Inviata
**Mittente**	Ministero della Salute (10128)
**Destinatario**	Centro Cardiologico S.P.A. Fondazione Monzino (10075)
**Progetto**	RCR-2022-23682288 - Rete CARDIO - Integrated strategies for the study of tissue and molecular determinants of vulnerable atherosclerotic plaque - Procedura nota DGRIC n. 1401 del 13/04/2022 Fondo progetti reti EF 2022
**Tipo**	Avvio progetto
**Oggetto**	Avvio progetto 1/1/2022 al 30/06/2023 - Richiesta cronoprogramma/emissione attestazione di credito pari al 60% del finanziamento assegnato
**Messaggio**	Tenuto conto delle indicazioni di cui alla nota in allegato, si chiede di trasmettere, entro il 5 ottobre 2022, con gli appropriati messaggi:<br>1) Cronoprogramma attività e relativo stato attuativo firmato dal PI (modello allegato)<br>2) Attestazione di credito e dichiarazione di impegno (nelle forme e nei modi del DPR 445/2000) a erogare la rata di acconto alle UO partecipanti, pari a € 1.036.812,00 (in una cartella compressa)<br><br>Il Direttore dell'Ufficio 5 - DGRIC dr.ssa Giselda SCALERA<br>Referente dr.ssa Dafne Verongalli

**Allegato**	22_RCR fondi 2022_rettifica.7z

Tipo messaggio	Messaggio proattivo
Data inizio progetto	01/01/2022
