# Supplementary material for: miRNome profile in blood samples upstream and downstream of the coronary lesion and arterial aortic root before and after angioplasty in subjects with chronic and acute coronary syndrome: A pilot observational study protocol (Plaque study)
Source: PLoS One. 2025 Jun 13;20(6):e0324467. doi: 10.1371/journal.pone.0324467 (PMC12165368; doi:10.1371/journal.pone.0324467)
Supplement: S3 File — (PDF) [file pone.0324467.s003.pdf]

Identificazione di biomarcatori innovativi circolanti e cellulari in soggetti con sindrome coronarica acuta e cronica

Studio pilota nell'ambito del progetto finanziato dal Ministero della Salute, fondo ricerca corrente Reti IRCCS 2022: Integrated strategies for the study of tissue and molecular determinations of vulnerable atherosclerotic plaque (RCR-2022-23682288)

Principal Investigator:
Gabriele Gabrielli
Responsabile UO Cardiologia Interventistica
IRCCS INRCA
Ancona

Team di ricerca:

- Roberto Antonicelli, direttore medico, UO Cardiologia e Telemedicina, IRCCS INRCA Ancona
- Olga Protic: biologo, UO Cardiologia, IRCCS INRCA, Ancona
- Anna Rita Bonfigli: biologo, Clinical Trial Office, Direzione Scientifica, IRCCS INRCA, Ancona
- Fabiola Olivieri: biologo, Laboratorio analisi IRCCS INRCA, Ancona; Dipartimento di Scienze Cliniche e Molecolari, Università Politecnica delle Marche, Ancona

Sommario

Sinossi 3
Background 4
Scopo dello studio 4
Popolazione 4
Criteri d’inclusione/esclusione 5
Criteri di inclusione: 5
Criteri di esclusione 5
Procedura coronarografia/angioplastica e modalità di “stoccaggio” campioni biologici 5
Esame biomarcatori innovativi 6
Analisi statistica 7
Variabili raccolte 7
Rischi/benefici della partecipazione allo studio 7
Aspetti etici 7
Copertura Assicurativa 8
Gestione e notifica di eventi avversi/reazioni avverse 8
Piano di divulgazione e comunicazione dei risultati 9
Finanziamento 9
Bibliografia 9

Sinossi

Scopo dello studio	Scopo principale è valutare il livello di biomarcatori innovativi nella patogenesi dell'IMA, con particolare riferimento a quelli coinvolti nei processi infiammatori/immunitari.
Popolazione	In questo studio pilota, saranno arruolati 20 pazienti: 10 pazienti con Sindrome Coronarica Acuta (SCA) NSTEMI e 10 pazienti con Sindrome Coronarica Cronica (SCC), con indicazione clinica ed anatomia favorevole per angioplastica coronarica (PCI).
Criteri di inclusione	➢ Età > 18 anni<br>➢ Indicazione clinica alla procedura PCI secondo le ultime Linee Guida ESC (European Society of Cardiology)<br>➢ Le stenosi coronariche trattate con angioplastica devono essere localizzate nei segmenti prossimali dei tre vasi principali: IVA (arteria interventricolare anteriore), CX (arteria circonflessa) e CD (arteria coronaria destra)<br>➢ I vasi interessati devono avere un diametro del loro tratto medio-distale ≥ 3 mm<br>➢ presenza di consenso informato scritto<br>➢ Pazienti con SCA-NSTEMI ed indicazione clinica ed anatomia favorevole per PCI (*per il gruppo SCA*)<br>➢ Pazienti con SCC decorrente clinicamente con Angina Stabile (o significativo equivalente anginoso) ed indicazione clinica con anatomia favorevole per PCI *(per il gruppo SCC*)
Criteri di esclusione	➢ Controindicazioni a terapia anticoagulante/antiaggregante<br>➢ Estese calcificazioni e/o tortuosità dei segmenti epicardici maggiori<br>➢ Evidenza di occupazione trombotica<br>➢ Pazienti con instabilità emodinamica<br>➢ Pazienti con FE (frazione di eiezione) < 35 %<br>➢ Pazienti con IRC di grado severo (e-GFR < 30 mL/min)
Biomarcatori innovativi	Dai seguenti prelievi:<br>➢ prima e dopo angioplastica venoso periferico<br>➢ prima e dopo angioplastica a monte e a valle della lesione coronarica<br>➢ prima e dopo angioplastica prelievi arteriosi a livello della radice aortica<br>verrà separato il siero, che verrà aliquotato e congelato a -80°C fino alle analisi successive.<br>Sul siero verrà analizzata l'espressione dei microRNA (molecole di RNA a filamento singolo). In particolare verranno dosati microRNA correlati all'infiammazione, quali miR-146a e miR-21, e microRNA espressi in maniera più specifica dai cardiomiociti, quali il miR-499, nelle varie tipologie di prelievi di pazienti affetti da sindromi coronariche acute o croniche. Verrà effettuata anche l'analisi quantitativa di molecole pro ed antiinfiammatorie quali IL-6, IL-33, sST2, IL-10. Verrà inoltre effettuata la misurazione dei prodotti finali della glicazione avanzata (AGE) e delle forme solubili del recettore AGE (sRAGE).
Statistica	Verranno confrontati i valori di espressione dei microRNA circolanti e delle molecole pro ed antiinfiammatorie nei prelievi arteriosi e venosi, prima e dopo la procedura interventistica, mediante test per misure appaiate. Inoltre saranno confrontati i livelli dei biomarcatori tra i due gruppi di pazienti (gruppo SCA e gruppo SCC) per le varie tipologie di prelievo mediante test per il confronto tra campioni indipendenti. Un valore di $p<0.05$ sarà considerato statisticamente significativo.

Background

Nonostante decenni di studi molto approfonditi a tutt'oggi permangono molti aspetti da chiarire nella fisiopatologia dell'Infarto Miocardico Acuto (IMA). In particolare rimangono da chiarire quei meccanismi che portano una placca non solo ad instabilizzarsi, fenomeno molto frequente e comune, ma soprattutto ad innescare quei processi coagulativo/trombotici che generano la Sindrome Coronarica Acuta. Poiché il substrato anatomico delle coronarie relativamente spesso tra una coronaropatia cronica ed acuta non presenta particolari differenze nell'entità della stenosi, abbiamo deciso di utilizzare un modello che s'incentra su di una base «anatomica» apparentemente comune. Si provvederà ad arruolare pazienti affetti da Sindrome Coronarica Acuta (SCA) e Sindrome Coronarica Cronica (SCC) con indicazione clinica alla coronarografia che eventualmente dimostri la presenza di coronaropatia significativa in entrambe questa tipologia di pazienti. Si provvederà pertanto a prelevare, oltre al sangue periferico, sangue coronarico ed eventuali frammenti di placca per evidenziare eventuali differenze nei biomarcatori esaminati (con particolare riferimento al profilo immunitario/infiammatorio).

Il ruolo di diversi miRNA nella regolazione di diversi percorsi biologici coinvolti nello sviluppo e nella progressione dell'aterosclerosi è stato descritto utilizzando modelli preclinici. Nell'uomo è stata identificata un'espressione alterata di diversi miRNA circolanti in presenza di placche aterosclerotiche stabili. Il nostro gruppo di ricerca ha precedentemente identificato i microRNA circolanti coinvolti nella modulazione dello stato infiammatorio, gli inflammamiR, nonché di microRNA correlati in maniera più specifica alla necrosi dei cardiomiociti (ad esempio miR-499). Tuttavia, per quanto ci è dato sapere, non ci sono dati pubblicati sulla relazione tra l'espressione di microRNA nel sangue coronarico, arterioso (aorta) e venoso periferico di pazienti affetti da sindromi coronariche acute o croniche. Il protocollo includerà anche la misurazione dei prodotti finali della glicazione avanzata (AGE) e delle forme solubili del recettore AGE (sRAGE) nel sangue dei pazienti arruolati, in quanto queste molecole giocano un ruolo chiave nell'induzione di processi infiammatori.

Scopo dello studio

Scopo principale è valutare il livello di biomarcatori innovativi nella patogenesi dell'IMA, con particolare riferimento a quelli coinvolti nei processi infiammatori/immunitari. L'evidenza di significativi cambiamenti in uno o più dei parametri esaminati tra le diverse forme di Sindrome Coronarica porterebbe un notevole contributo per comprendere la possibile patogenesi dell'IMA.

Popolazione

In questo studio pilota, saranno arruolati 20 pazienti: 10 pazienti con Sindrome Coronarica Acuta (SCA) NSTEMI e 10 pazienti con Sindrome Coronarica Cronica (SCC), con indicazione clinica ed anatomia favorevole per angioplastica coronarica (PCI).

Criteri d'inclusione/esclusione

Criteri di inclusione:

- Età > 18 anni
- Indicazione clinica alla procedura PCI secondo le ultime Linee Guida ESC (European Society of Cardiology)
- Le stenosi coronariche trattate con angioplastica devono essere localizzate nei segmenti prossimali dei tre vasi principali: IVA (arteria interventricolare anteriore), CX (arteria circonflessa) e CD (arteria coronaria destra)
- I vasi interessati devono avere un diametro del loro tratto medio-distale ≥ 3 mm
- presenza di consenso informato scritto
- Pazienti con SCA-NSTEMI ed indicazione clinica ed anatomia favorevole per PCI (*per il gruppo SCA*)
- Pazienti con SCC decorrente clinicamente con Angina Stabile (o significativo equivalente anginoso) ed indicazione clinica con anatomia favorevole per PCI *(per il gruppo SCC*)

Criteri di esclusione

- Controindicazioni a terapia anticoagulante/antiaggregante
- Estese calcificazioni e/o tortuosità dei segmenti epicardici maggiori
- Evidenza di occupazione trombotica
- Pazienti con instabilità emodinamica
- Pazienti con FE (frazione di eiezione) < 35 %
- Pazienti con IRC di grado severo (e-GFR < 30 mL/min)

Procedura coronarografia/angioplastica e modalità di "stoccaggio" campioni biologici

L'esame coronarografico verrà effettuato con accesso arterioso radiale o femorale, utilizzando introduttori e cateteri da 6 o da 7 F, ed eseguito con angiografo Siemens Artis. Verrà effettuata inoltre l'indagine ultrasonografica intracoronarica (IVUS), che viene effettuata di routine nel nostro Centro in caso di angioplastiche complesse, mediante sistema Core Mobile Philips, che consentirà di effettuare la valutazione della placca sia con la modalità scala di grigi che con quella Virtual Histology.

La procedura di angioplastica verrà effettuata secondo la pratica corrente.

Prelievi ematici e, se possibile, di frammenti di placca/trombo, in prossimità (< 10 mm, a monte e a valle) della lesione coronarica verranno effettuati prima e dopo l'angioplastica mediante microcatetere Pronto LP 5 F (Teleflex Inc. USA).

Il sistema di protezione distale utilizzato per raccogliere frammenti di placca durante la procedura interventistica è il Sistema di protezione embolica Spider FX (Medtronic Europa); il sistema è costituito da un microcatetere con filo guida alla cui estremità è fissato un cestello in nitinol, di misura variabile, e verrà posizionato, distalmente alla lesione da esaminare, all'inizio della procedura interventistica, consentendo di raccogliere, e infine recuperare, gli eventuali frammenti di placca che si dovessero dislocare durante la procedura, senza interferire con l'apporto di sangue alla periferia del vaso, vista la pervietà del filtro; alla fine della procedura il filtro verrà rimosso e i frammenti di placca potranno essere recuperati. Il materiale raccolto nel cestello del dispositivo Spider Fx verrà accuratamente rimosso dal filtro, raccolto in provette tipo Falcon e congelato a -80 gradi per future analisi presso il laboratorio analisi del POR INRCA di Ancona.

Verranno inoltre effettuati prelievi arteriosi a livello della radice aortica, prima e al termine dell'angioplastica, mediante catetere diagnostico JR4 Super Torque Plus 5 F (Cordis Inc. USA), e prelievi venosi a livello del sangue periferico, prima e al termine dell'angioplastica.

Esame biomarcatori innovativi

Dai seguenti prelievi (ognuno circa 1 mL di sangue):

- prima e dopo angioplastica venoso periferico
- prima e dopo angioplastica a monte e a valle della lesione coronarica
- prima e dopo angioplastica prelievi arteriosi a livello della radice aortica

verrà separato il siero, che verrà aliquotato e congelato a -80 gradi presso il laboratorio analisi del POR INRCA di Ancona fino alle analisi successive.

Sul siero verrà analizzata l'espressione dei microRNA (molecole di RNA a filamento singolo). In particolare verranno dosati microRNA correlati all'infiammazione, quali miR-146a e miR-21, e microRNA espressi dai cardiomiociti, quali il miR-499, nelle varie tipologie di prelievi di pazienti affetti da sindromi coronariche acute o croniche. Verrà effettuata anche l'analisi quantitativa di molecole pro ed antiinfiammatorie quali IL-6, IL-33, sST2, IL-10. Verrà inoltre effettuata la misurazione dei prodotti finali della glicazione avanzata (AGE) e delle forme solubili del recettore AGE (sRAGE).

I campioni biologici, compresi i frammenti di placca, saranno conservati, anche oltre il termine dello studio per massimo 10 anni, per eventuali ulteriori ricerche o sperimentazioni approvate dal Comitato Etico nell'ambito scientifico di indagine dello studio

Analisi statistica

Verranno confrontati i valori di espressione dei microRNA circolanti e delle molecole pro ed antiinfiammatorie nei prelievi arteriosi e venosi, prima e dopo la procedura interventistica, mediante test per misure appaiate. Inoltre saranno confrontati i livelli dei biomarcatori tra i due gruppi di pazienti (gruppo SCA e gruppo SCC) per le varie tipologie di prelievo mediante test per il confronto tra campioni indipendenti. Un valore di $p<0.05$ sarà considerato statisticamente significativo.

Variabili raccolte

Durante lo studio verranno raccolte informazioni sullo stato di salute dei soggetti partecipanti (diagnosi, terapie farmacologiche, esami diagnostici, dati biologici). Fattori di rischio comuni (fumo, alcool, movimento). Dati sociodemografici (età, sesso, scolarità, nazionalità). I dati clinici del paziente saranno raccolti dalla documentazione prodotta durante il percorso assistenziale tramite cartella clinica elettronica.

Rischi/benefici della partecipazione allo studio

Lo studio, seguendo le norme della buona pratica clinica e dell'appropriatezza nell'esecuzione dell'esame coronografico, non comporta nessun particolare cambiamento rispetto alla "normale" gestione clinica di questa tipologia di pazienti.
Il prelievo del materiale biologico comporta un breve "allungamento" nei tempi della normale procedura, verosimilmente non rilevante per quanto riguarda il comfort del paziente ed il rischio a cui è esposto.
Pertanto i benefici sono rappresentati dal notevole contributo che il paziente con la sua partecipazione può dare all'incremento della conoscenza della fisiopatologia dell'infarto miocardico i rischi sono sostanzialmente sovrapponibili a quelli di una normale procedura di coronarografia ed angioplastica che avrebbe in ogni caso dovuto fare secondo Linee Guida per il trattamento della sua patologia di base.

Aspetti etici

Lo studio sarà condotto in accordo alle Norme di buona pratica clinica, ai principi etici derivanti dalla Dichiarazione di Helsinki e dalla normativa vigente in materia di studi osservazionali.

Lo studio verrà condotto tenendo conto dei requisiti regolatori e degli adempimenti di legge. Esso sarà avviato a seguito dell'ottenimento della valutazione e dell'approvazione da parte del Comitato Etico indipendente e del completamento degli adempimenti amministrativi.

Inoltre:

- Tutti i soggetti potenzialmente eleggibili dovranno ricevere le informazioni complete sullo studio e fornire il proprio consenso alla partecipazione allo studio;
- I partecipanti potranno lasciare il progetto in qualsiasi momento se ritengono che la partecipazione sia troppo onerosa o si sentano a disagio per qualsiasi motivo.
- I partecipanti dovranno fornire il consenso al trattamento dei dati personali in forma anonima ed aggregata, ai sensi del Regolamento UE 2016/679 (GDPR) relativo alla protezione delle persone fisiche con riguardo al trattamento dei dati personali e al D. Lgs. N. 101/2018 – Disposizioni per l'adeguamento della normativa nazionale alle disposizioni del Regolamento Europeo 2016/679;
- Il soggetto viene informato e gli viene richiesto di fornire il consenso informato ad hoc per partecipare allo studio, inclusa la conservazione dei dati fino a 15 anni dalla conclusione dello studio.
- Al fine di salvaguardare il rispetto della privacy, i dati raccolti saranno mantenuti strettamente confidenziali e verrà allestito un database di proprietà dell'INRCA presso l'UO di Cardiologia dell'INRCA di Ancona contenente i dati dei soggetti coinvolti nello studio. Nel rispetto delle norme di protezione sarà previsto l'accesso, mediante password, sia per il server che per la sezione contenente il database, solo al personale delle U.O. coinvolte nello studio per l'inserimento e l'elaborazione dei dati.
- Titolare del trattamento è l'IRCCS INRCA, con sede in Via Santa Margherita 5 – 60124 – Ancona. Inoltre l'IRCCS INRCA, ai sensi dell'articolo 37 del GDPR EU 2016/679, ha proceduto ad individuare e nominare il Data Protection Officer (DPO) contattabile all'indirizzo dpo@morolabs.it
- I partecipanti saranno informati che i loro dati possono essere esaminati da personale autorizzato o da parte di membri del comitato etico competente e da funzionari delle autorità regolatorie competenti;

Copertura Assicurativa

Data la natura osservazionale dello studio proposto, non sono necessarie polizze assicurative aggiuntive rispetto a quelle già previste per la normale pratica clinica. E' comunque presente la polizza assicurativa dell'Istituto (AMTRUST INSURANCE AGENCY ITALY SRL polizza n. RCH20020000008, scadenza 30/06/2023).

Gestione e notifica di eventi avversi/reazioni avverse

Essendo lo studio di natura osservazionale non sono prevedibili eventi/reazioni avverse tranne quelle presenti nelle procedure di pratica clinica compatibili con la natura dello studio che fanno parte degli esiti registrati.

Piano di divulgazione e comunicazione dei risultati

Il responsabile scientifico dello studio s'impegnerà nella stesura di un rapporto finale ed a rendere pubblici i risultati al termine dello studio. I dati saranno resi pubblici in modo anonimo e presentati per quanto richiesto in modalità aggregata.

Finanziamento

Lo studio è finanziato dal Ministero della Salute, tramite il meccanismo della Ricerca Corrente di Rete Cardiologica (RCR-2022-23682288).

Bibliografia

- Angelini A. et al, Distal Protection With a Filter Device During Coronary Stenting in Patients With Stable and Unstable Angina, *Circulation* 2004;110:515-521
- Mayer W et al., Inflammatory Markers at the Site of Ruptured Plaque in Acute Myocardial Infarction, *Circulation* 2005;111:1355-1361
- Nishio M. et al., Association of Target Lesion Characteristics Evaluated by Coronary Computed Tomography Angiography and Plaque Debris Distal Embolization During Percutaneous Coronary Intervention, *Circ J* 2014; 78: 2203–2208
- Olivieri F. et al., Admission levels of circulating miR-499-5p and risk of death in elderly patients after acute non-ST elevation myocardial infarction. *Int J Cardiol*. 2014; 15;172(2):e276-8.
- Olivieri F. et al., miR-21 and miR-146a: The microRNAs of inflammaging and age-related diseases. *Ageing Res Rev.* 2021 Sep; 70:101374.
- Olivieri F. et al., Circulating microRNAs (miRs) for diagnosing acute myocardial infarction: an exciting challenge. *Int J Cardiol*. 2013 Sep 10;167(6):3028-9.
- Robertson L. et al., Release of protein as well as activity of MMP-9 from unstable atherosclerotic plaques during percutaneous coronary intervention, *Journal of Internal Medicine* 2007; 262; 659–667
- Sabbatinelli J. et al., Circulating levels of AGEs and soluble RAGE isoforms are associated with all-cause mortality and development of cardiovascular complications

in type 2 diabetes: a retrospective cohort study. Cardiovasc Diabetol. 2022 Jun 6;21(1):95.

- Savchenko AS et al., Expression of pentraxin 3 (PTX3) in human atherosclerotic lesions, *J Pathol* 2008; 215: 48–55
